# Construction, Control, and Application of Cyborg Animal Composed of Biological and Electromechanical Systems

**DOI:** 10.34133/cbsystems.0486

**Published:** 2026-03-26

**Authors:** Yue Ma, Chuang Zhang, Fei Nie, Hengshen Qin, Qi Zhang, Yiwei Zhang, Lianchao Yang, Lianqing Liu

**Affiliations:** ^1^ State Key Laboratory of Robotics and Intelligent Systems, Shenyang Institute of Automation, Chinese Academy of Science, Shenyang 10016, P. R. China.; ^2^ University of Chinese Academy of Sciences, Beijing 100049, P. R. China.

## Abstract

The limitations of biohybrid and mechanical robots, including insufficient control accuracy, limited flexibility, long-term stability, and endurance, have spurred considerable research interest in cyborg animals, which leverage the innate locomotion capabilities, physiological systems, and natural intelligence of organisms to perform tasks with high adaptability, superior performance, and extended endurance. This study provides a comprehensive overview of cyborg animals within the framework of animal taxonomy, summarizing the current state of research from a zoological perspective. Subsequently, the effect of different control techniques on the locomotion performance of cyborg animals was examined, with a special emphasis on 2 prominent research areas: brain–computer interfaces and muscle-receptor electrical stimulation. In addition, the role of advances in electronic backpack design and navigation control algorithms in enabling closed-loop control and applications, including swarm robotics, environmental exploration, and human–machine interaction, is also introduced, offering valuable insights for developing cyborg animals. This study highlights 4 critical aspects essential for the future advancement of cyborg animals by synthesizing recent progress and clarifying technical distinctions: adaptation between control strategies and animals, biocompatibility and stability of electronic backpacks, construction of interactive hybrid robotic systems, and ethical and welfare considerations related to the experimental animals, with the hope of facilitating the optimization and application of cyborg animal systems.

## Introduction

**Fig. 1.**
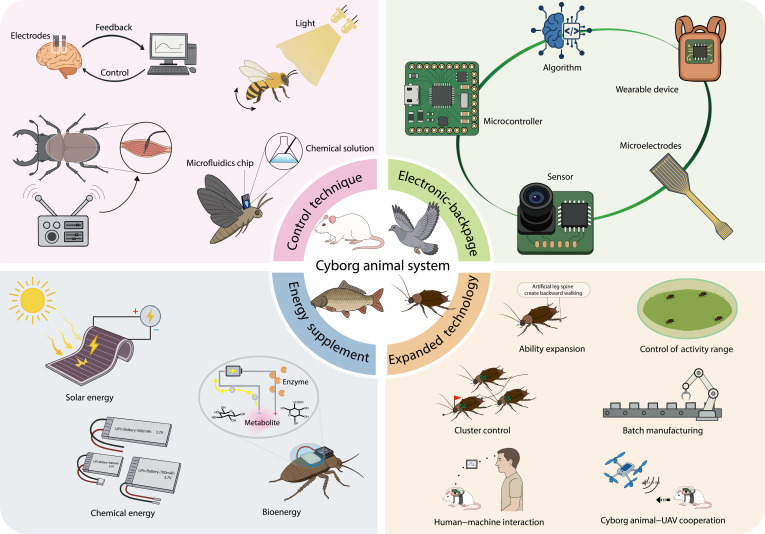
Overview of the cyborg animal system.

Close to the 20th century and with the rapid advancement of computer technology, the intelligence levels of traditional silicon-based robots improved markedly, and their morphological architectures exhibited a trend toward diversification. However, traditional silicon-based robots rely predominantly on rigid mechanical structures, utilizing batteries as energy sources and electric drive components as the foundation for sensing and motion (Fig. 1). Advanced technologies such as machine learning and intelligent decision-making can be integrated into robots to mitigate some of these defects; however, their mobility, autonomous decision-making capabilities, and endurance remain markedly inferior to those of living animals [1,2]. In addition, the adaptability of bionic robots to complex environments, autonomous decision-making in emergencies, and interaction capabilities with external objects are far less than those of real animals. Consequently, researchers progressively shifted their focus from biorobots to biohybrid robots. The biohybrid robots represent a novel type of integrated robot that deeply fuses living materials with mechanical structures, achieving comprehensive physical and informational integration of living–mechanical systems while unifying the strengths of both biological and mechanical systems [3].

Cyborg animals, a critical branch of biohybrid robots, integrate machine intelligence with biological intelligence and leverage the inherent perception, movement, and energy supply mechanisms of living animals through external artificial stimulation for controlling the animals to execute human instructions. Cyborg animals can exploit the advantages of living animals, such as strong decision-making, extended endurance, high effectiveness, and superior adaptability, while incorporating control strategies and mission planning into the operational loop to act more effectively and controllably [4]. In addition, they can act far more intelligently and flexibly with longer endurance and powerful self-healing ability than fully mechanical robots in complex environments. Cyborg animals suffer from persistent challenges. For example, achieving consistency not only among similar or the same species of animals but also in experiments with the same animal is difficult. In addition, the stimulating control effect is also easily and obviously impacted by the neural signals of the animal or environmental upheaval. This increases the difficulty of control and control system portability and can only maintain control during a short stimulation duration. Thus, the controllability and application of these robots are not as good as those of mechanical robots. In 1997, Holzer and Shimoyama [5] achieved artificial control over the movement of a live insect for the first time using electrical stimulation to guide cyborg cockroaches along a straight path. This pioneering study marked the formal inception of cyborg animal research. Early studies in this domain focused on small insects [6] and rats [7]. With continuous advancements in cyborg animals, the range of target species has expanded to birds, fish, and other animals. Further, control methods have diversified, encompassing brain–machine interface control, electrical stimulation of muscles or sensory organs, and chemical stimulation via hormones.

In this study, we organize and analyze typical references with keywords including “animal robot”, “cyborg insect”, and “biohybrid robot” because of the dispersed and inconsistent keywords of this research area. We also analyze some articles with high correlations, such as “electronic backpack” and “brain–machine interface”, for the discussion on robot construction and control. This review provides 2 special perspectives for understanding this area distinctly: animal taxonomy and cyborg animal construction. The first summarizes the research state through experimental animal species to provide a clear and consistent recognition of the whole area, and the second enables researchers to quickly obtain the basic procedure and advanced techniques for constructing cyborg animals. In addition, we summarize 4 critical points essential for area advancement: adaptation between the control strategy and animals, biocompatibility and stability of electronic backpacks, construction of interactive hybrid robotic systems, and ethical and welfare considerations related to experimental animals, expecting to promote further development of this area.

## Research on Cyborg Animals Based on Animal Taxonomy

Each of the wide variety of animal species in nature has distinct forms and characteristics; thus, studies on cyborg animals always show low similarity. Consequently, classifying and summarizing the existing studies on this topic based on animal taxonomy is necessary. Table 1 gives a comparison of several key performance of cyborg vertebrates.

**Table 1.** Comparison of the performance of cyborg vertebrates.

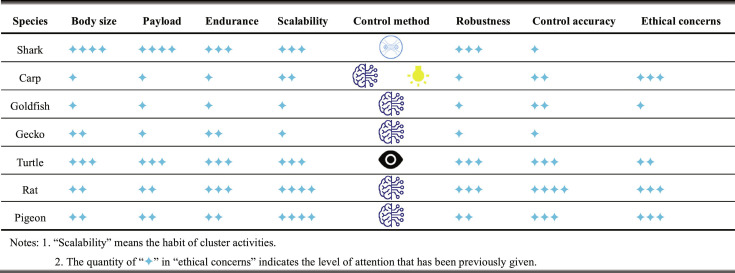

### Vertebrate phylum

Vertebrates are characterized by the presence of a notochord, dorsal nerve cord, and pharyngeal slits at a certain developmental stage [8]. This phylum includes fish, reptiles, birds, and mammals; their highly developed brains and concentrated sensory organ systems endow them with strong information processing capabilities and diverse decision-making and movement behaviors. Consequently, brain–computer interfaces (BCIs) and some techniques related to organs have become the primary choice for exploring various locomotion and reaction capabilities. Further, vertebrates are subject to strict ethical regulations in many countries because they are living beings with the ability to perceive rather than only being used as experimental tools.

#### Class Pisces

Fish in the class Pisces show high flexibility and adaptation in underwater exploration and can be acquired easily, studied, and controlled. They rely on the cerebellum and midbrain in the brain region to control paired and unpaired fins for maintaining body balance and agility. Recently, fish-based experimental subjects that have achieved drive control include cyborg sharks, cyborg carp, and cyborg goldfish.

The shark lateral line system includes a lateral line to detect mechanical stimuli, an ampulla of Lorenzini for detecting electrical stimuli, and electroreceptors on the head sensitive to voltage gradients, enabling sharks to detect weak bioelectric fields of their prey [9]. Based on these findings, researchers achieved the remote control of cyborg sharks through brain electrical stimulation and vibration stimulation of the lateral line [10]. Further, they induced the directional movement of *Sphyrna lewini* by simulating bioelectric fields [11]. The body movements of carp and goldfish are rhythmically controlled by central pattern generators (CPGs), and the activation of CPGs and the initiation and maintenance of movement require an excitation drive from the brain [12]. Therefore, stimulating the medial longitudinal fasciculus nucleus can achieve the controlled movement of fish [13,14]. Peng et al. [15] constructed a noninvasive optically controlled cyborg carp.

#### Class Reptilia

Reptiles have 5-toed limbs, and the bones of the extremities have a right-angled relationship with the axial skeleton. Among these, geckos and turtles crawl by alternating the movements of their 4 limbs [16]. The excellent rock-climbing ability and special foot structure of geckos have attracted considerable attention; however, simultaneous fine toe movements and multilimb coordination control are difficult. Adsorption crawling based on microscopic force cannot be achieved, which leads to insufficient research progress. Research has shown that the *Gekko gecko* can achieve toe detachment, movement, and adhesion through toe movements and perform specific toe movements after electrical stimulation; however, this is only an in vitro experiment and does not constitute actual movement functions [17]. Coordinate crawling with 4 limbs is achieved by electrically stimulating the midbrain tegmentum (MBT) of geckos and combining 3 movement patterns: spinal deformation, limb adduction, and extension, achieving limited turning and forward movement [18,19].

Lee et al. used turtles as research subjects and achieved guiding control using 2 methods: virtual obstacle avoidance stimulation [20,21] and virtual cue stimulation [22]. These 2 methods utilize the spontaneous behavior of turtles without any surgery or additional sensors. The lack of research on turtles can be attributed to the instability of the noninvasive control method and difficulty of performing surgery in the presence of a carapace.

#### Class Mammalia

Mammals are adapted to almost all ecological environments and are the most structurally complete, behaviorally and functionally complex, and adaptable group among vertebrates [16]; their high-level intelligence, ability to perform agile and rapid movements, excellent learning and recovery abilities, and adaptability represent abundant possibilities and research value. Rats are the optimal choice for animal experiments given the solid foundation in biological and physiological research as well as the scientifically standardized basis for cultivating them. Meanwhile, BCI-control-based rats can provide theoretical and engineered clinical verification for BCI application in humans because of their similar brain structures and functions.

Control modes used to induce rat movement via BCI can be divided into those based on sensory brain stimulation and motor brain stimulation. The sensory brain region directly triggers reflexes or drives motivational behaviors through the “sensory–motor transformation” mechanism, which relies on the sensory–motor cortex connection and the thalamus–limbic pathway. Forward movement and turning in rats can be controlled by stimulating brain regions with position and movement perception functions and those involved in reward, motivation, and pleasure processing [23–25]. The motor-controlled brain region converts motor intentions into specific actions through hierarchical and coordinated neural circuits. Actions such as turning and stopping can be achieved by stimulating related brain regions [26,27]. A combination of ultrasound, electrical, and optical stimulations can achieve remote control [28]. Through the combined stimulation of multiple brain regions and integration of navigation algorithms and sensors, a closed-loop control system can enable precise motion control [29], autonomous positioning [30], intelligent navigation [31–33], and autonomous search [34] in small mazes or indoors.

#### Class Aves

Birds can soar freely because of their unique multiporous bones, wings, and tail feathers; thus, they have distinctive advantages in terms of spatial perception and aerial reconnaissance. Pigeons have become the main research subjects for avian robots because of their ease of breeding, stable flight performance, low energy consumption, and strong navigation and spatial cognition abilities [35,36]. Different electrical signals can be applied to different neural points in the brain to achieve movement control because the process from receiving stimulation to exhibiting motor behavior involves multiple levels of the pigeon nervous system. Brain regions related to fear generation [37–40] and emotion regulation [41–44] can be used to induce contralateral turning, locomotion freezing, ipsilateral lateral movement, and takeoff by flapping wings. Graded and quantified control of pitch attitude and turning angle adjustment can be achieved through the stimulation of behavioral instructions and motor coordination in the brain regions [45–48]. As indoor experiments cannot reflect the true locomotion control level of the flight ability of cyborg pigeons, outdoor controlled remote flight experiments with onboard GPS (Global Positioning System) modules have also been launched, and the stimulation parameters and corresponding location change of cyborg pigeons can be recorded and transmitted [49]. Involved with the barometric pressure sensor, altitude messages can be collected and controlled [48]. In addition, a closed-loop control system based on the GPS and automatic stimulation of cyborg pigeons according to their real-time trajectory deviations can achieve the function of controlling the pigeons to fly along a preplanned path [50].

### Invertebrate phylum

#### Arthropoda phylum

Arthropods are characterized by bilateral symmetry and highly specialized segmented bodies, and the elasticity of their exoskeletons stores energy for activities such as wing flapping and jumping. The segmentation of appendages enhances movement flexibility and has certain functions in sensing, feeding, and moving [16]. Recently, arthropod robots have become the focus of research involving moths, beetles, cockroaches, locusts, bees, spiders, and dragonflies. The small size and relatively simple muscle–behavior association mechanism of these insects make noninvasive and direct muscle-receptor stimulation methods the primary choices. Beetles and cockroaches exhibit high physiological resilience, fast reproductive rates, ingenious locomotion behaviors, and simple body structures. Beetles such as *Mecynorrhina torquata* have both flight and crowing abilities to achieve amphibious operation, which makes them conducive to achieving cluster control and other attempts. Table 2 shows a recapitulative comparison about the performance of cyborg invertebrates.

**Table 2.** Comparison of the performance of cyborg invertebrates.

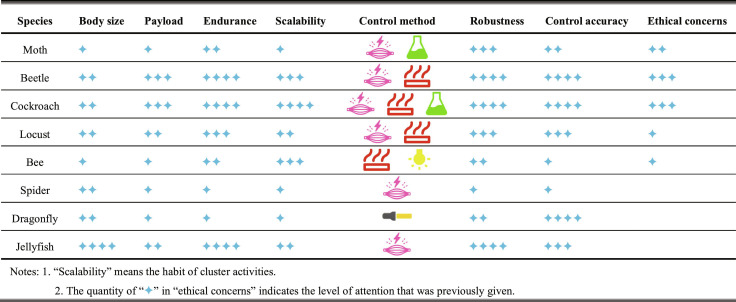

In 2007, Bozkurt et al. achieved flight turning [51], initiation and termination [52,53], and yaw flight [54] control of the cyborg *Manduca sexta* in a tethered state based on early metamorphosis insertion technology (EMIT). Further, they achieved yaw flight control and rapid take-off via light stimulation [55] and thermal control [56]. Hinterwirth et al. [57] demonstrated flight pitch control via antennal muscle stimulation, while Erickson et al. [58,59] used microfluidic drug delivery and antennal lobe (AL) stimulation to modulate flight speed.

Cyborg beetles have been studied extensively for their ability to control flight and crawl via electrical stimulation. In terms of flight control, Sato et al. achieved a hierarchical control of flight direction, altitude, and speed by applying electrical stimulation with different parameters to the basalar muscle [60], subalar muscle (SM) [61,62], and third axillary (3Ax) muscle [63]. Compound vision and brain stimulations were combined to control flight initiation and termination [64–66] and achieved precise flight control [67–69]. For crawling control, leg muscle (LM) stimulation enables the gait, step length, and speed to be modulated [70–72]. The walking direction and backward movement control are induced via antennal stimulation [73], lateral movement is achieved via elytral stimulation [74,75], and closed-loop gait regulation can be achieved via feedback control algorithms [76,77]. In addition, low-temperature thermal stimulation of the antenna can induce turning behavior in cyborg beetles [52,53]. Based on the realization of forward movement, the turning and hierarchical control of cyborg cockroaches through the electrical stimulation of antennae (AT) and cameras [78–80], the combination of camera and computer vision tracking technology [81], or onboard camera and GPS active antenna chipset sensors [82–84] can achieve positioning and wireless closed-loop control, while inertial measurement units (IMUs) can achieve autonomous obstacle avoidance exploration [85,86]. Combining electrical stimulation with methyl salicylate chemical stimulation can enhance the environmental exploration ability of a robot [87].

Cyborg locust jumping control can be modulated via the electrical stimulation of hind legs [88,89], AT, and cercus [90], while thermal stimulation [91] and optical stimulation [92] can control the behavior of locusts. In addition, the flight states of bumblebees [93–95] and dragonflies [96] were adjusted through different light stimulations, and the multileg coordinated drive of a spider [97] was controlled through muscle stimulation. However, these will not be extensively discussed in subsequent chapters because there is a shortage of research recordings and comparison evidence.

#### Cnidaria phylum

The cyborg jellyfish remains the only experimentally validated cyborg cnidarian because of its low intelligence and adaptation, weak physical and locomotion ability, and lack of deep-sea detection, control, communication, and positioning of cnidarians. Its propulsion mechanism relies on muscular contractions that expel water from the subumbral cavity to generate thrust, supplemented with energy recapture and suction propulsion strategies [98]. Based on these biomechanical principles, Xu et al. [99,100] developed a cyborg jellyfish employing electrically stimulated muscular control, achieving rapid forward locomotion with minimal energy expenditure. Owaki et al. [101] achieved coherent and predictable swimming control by investigating self-organized criticality and made locomotion prediction possible through machine learning. This paper focuses on the comparison and discussion between robot construction and control. Although the cyborg jellyfish has the ability to explore the ocean, the uniqueness of its body structure, muscle types, and neural control networks makes it distinct from other animals; therefore, it will not be discussed in the following chapters.

Despite different animals, the control technique utilization and preference are also discrepant, and the control paradigms can be categorized from a technical perspective.

## Locomotion Control Techniques for Cyborg Animals

Cyborg animal experiments select animals with a certain intelligence, fully developed or potentially fully developed neuromuscular structures, and those that possess certain movement skills. The behavior of animals is controlled by stimulating brain nerves or muscles for generating electrical signals, or through vision, avoidance, or other means [16].

### BCI for Stimulating Brain Regions

The BCI technique enables bidirectional interaction between the brain and machines without peripheral nerves and muscle output pathways by recording and decoding neural activities in the brain and converting them into instructions to control devices or by transmitting feedback information to brain regions through neural stimulation [102]. Brain regions related to behavior and locomotion include the cortex, basal ganglia, thalamus, midbrain, hypothalamus, neuromodulatory, and limbic regions. Escape behavior and contralateral turning occur when the brain region related to emotions such as fear or disgust is stimulated, and when the region related to motor coordination and behavioral control is stimulated, other behaviors such as forward walking can be induced. Navigation and tracking movements are achieved through the alternate stimulation of different brain regions with different reactions.

**Fig. 2.**
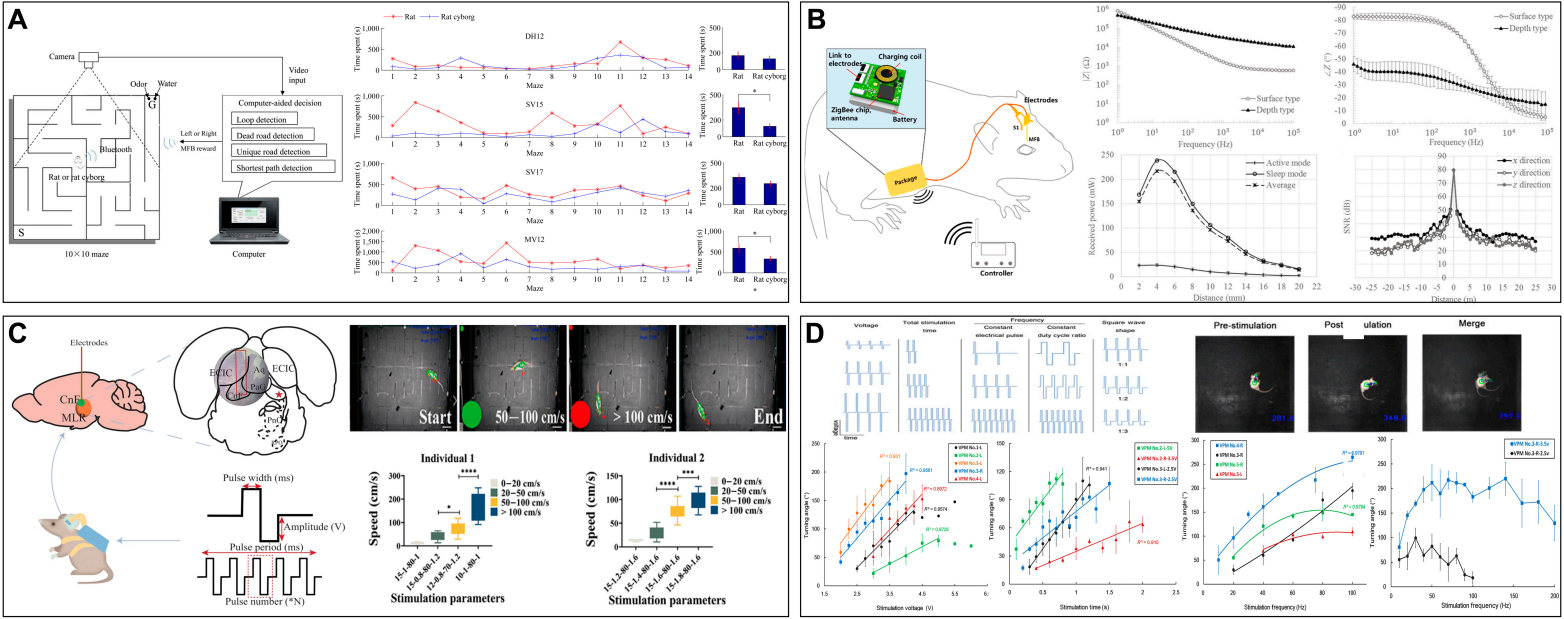
The performances of cyborg rats under different brain–computer interface control. (A) Electrical stimulation of MFB can induce cyborg rat to navigate in maze; several contrast experiments shows the exploration difference between rat and cyborg rat. Reproduced with permission [32]. Copyright 2016 PLOS. (B) A fully implantable neural stimulator can induce navigation movement in a cyborg rat in a simple maze through stimulation of S1 and MFB regions. Reproduced with permission [25]. Copyright 2019 MDPI. (C) Electrical stimulation of CnF can complete the speed-graded control of cyborg rats. Reproduced with permission [27]. Copyright 2024 Springer Nature. (D) The different stimulations with different parameters on VPM can lead to different behaviors. Reproduced with permission [24]. Copyright 2016 Elsevier.

As a standard laboratory animal that has been studied thoroughly, rats are low-cost and technologically mature BCI-controlled cyborg animals owing to their rich behavioral models and mature surgical procedures, moderate speed and load capacity, and nervous system structure and function comparable to that of humans. Research on cyborg rats began with a combination of the electrical stimulation of the primary somatosensory cortex (S1) and the medial forebrain bundle (MFB) of the reward and motor regulation pathways, where unilateral S1 stimulation for turning caused by virtual tactile sensation (Fig. 2B) [25] and MFB stimulation for behavioral reinforcement reward because of the increase in dopamine levels serve as a feedback reward for correct behavior (Fig. 2A) [32]. Further, MFB stimulation can also enable robots to autonomously navigate through 3-dimensional (3D) terrain [7,34]. Combined with an onboard visual recognition module, closed-loop autonomous navigation can be achieved through infrared target detection and a finite-state machine model; however, the success rate in the barrier field is only 65% [33]. In addition, Ling et al. [27] achieved graded control of a cyborg rat by stimulating the cuneiform nucleus of the mesencephalic locomotor region by adjusting stimulation parameters (Fig. 2C). Owing to the requirement of training in the S1 and MFB stimulation control strategies, control methods combining the stimulation of the ventral posterolateral (VPL) nucleus of the thalamus and amygdala nucleus (AMY) of the limbic system [23], stimulation of the ventral posteromedial nucleus (VPM) of the thalamus (Fig. 2D) [24], and stimulation of the nigrostriatal pathway (NSP) of the basal ganglia [103] have been proposed. VPL, AMY, and VPM stimulation target the thalamus, inducing turning through emotional or feeling pathways and achieving driving effects without training; deep brain stimulation methods enable more immediate control. These methods differ in terms of control dimensions and command semantics. The effect of VPL and AMY arise from the evasive turn triggered by “virtual punishment/aversion” and may cause irritability, while VPM stimulation offers more tangible and precise tactile cues within lower position fault tolerance. The stimulation of the NSP lead immediately turned to the opposite side, and 70 tests in the T-shaped maze were successful, while the time for determining the intersection decreased significantly [103]. As for the different control sites for BCI-controlled cyborg rats above, a similar electrical stimulation-behavior mapping mechanism enables them to have the possibility of mutual learning and integration. However, movement uniformity, multibehavior mixed exploration, and better consideration of animal health and ethical welfare remain as issues that must be addressed.

**Fig. 3.**
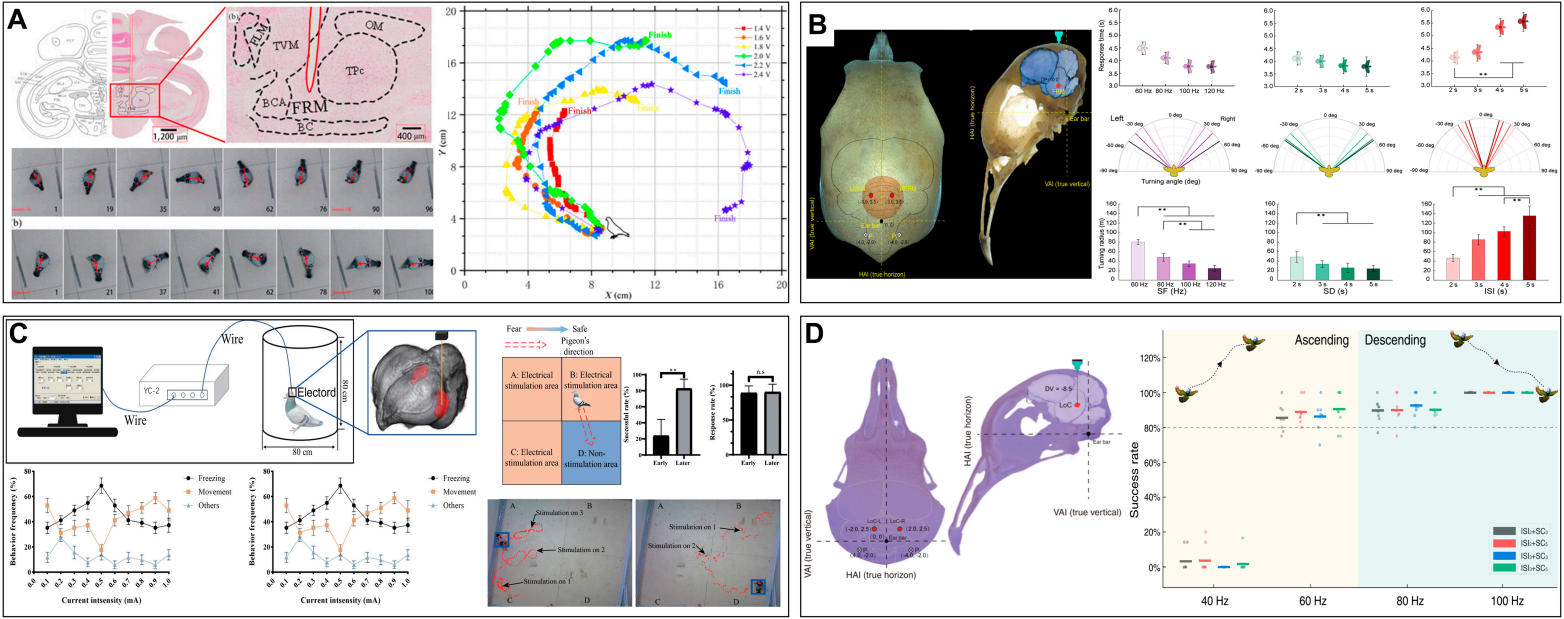
The locomotion and navigation of cyborg pigeons under different BCI stimulations. (A) Electrical stimulations of different voltages can lead to different flight turning of cyborg pigeons. Reproduced with permission [46]. Copyright 2024 MDPI. (B) The response time, turning angle, and turning radius of cyborg pigeon under the different stimulus parameters. Reproduced with permission [47]. Copyright 2023 Frontiers. (C) The cyborg pigeon can be reduced to move in a different direction through electrical stimulation of the SGP region because of feelings of fear. Reproduced with permission [40]. Copyright 2023 Springer Nature. (D) Different electrical stimulation parameters on LoC region can induce the flight altitude control of cyborg pigeon. Reproduced with permission [48]. Copyright 2025 AAAS.

The principle behind BCI-controlled cyborg pigeons is similar to that of cyborg rats, with the main differences being brain structure and locomotion abilities. Brain regions related to motor behavior, emotions, and motivation are important sources of behavioral initiation in pigeons. When the left or right dorsalis intermedius ventralis anterior of the thalamus is stimulated with electrical pulses, cyborg pigeons exhibit contralateral turning behavior opposite to the stimulation [37–39]. The stimulation of the posterior pallial amygdala of the limbic system induces ipsilateral lateral movement [41,42]. Owing to the simultaneous existence of displacement and turning angles, alternating stimuli on the left and right sides could enable the cyborg pigeon to move along a specified path [42]. Further, the electrical stimulation of the formatio reticularis medialis mesencephali of the midbrain can induce turning and circling behaviors (Fig. 3A) [45,46]. Adjustment of variables such as stimulation frequency and interval can achieve a certain degree of hierarchical control effect (Fig. 3B) [47]. The electrical stimulation of the nucleus intercollicularis that belongs to the midbrain can induce forward walking and flying behaviors [43,44]. The stimulation of the substantia grisea et fibrosa periventricularis (SGP) of the hypothalamus can pause all behaviors (Fig. 3C) [40]. In addition, the electrical stimulation of the locus coeruleus nucleus can regulate outdoor flight altitude without simultaneously affecting the flying direction, and the stimulation cycles are directly related to altitude (Fig. 3D) [48]. In addition, indoor and outdoor closed-loop control experiments were conducted using trajectory deviation feedback control methods [50] and GPS-based motion monitoring units [49]. As there are diverse stimulation sites to induce cyborg pigeons to fly and stop, the core consideration for selection and use is the region–behavior incidence relation pattern, in which a nucleus with clear functions, predictable behaviors, well-defined anatomical positioning, easy surgical implantation, and consistent and stable responses among different individuals may be given priority. The shortcomings include insufficient attention to surgical trauma and long-term health monitoring; difficulty in achieving continuous flexible navigation; insufficient success rate and path efficiency; shorter and lower pulses; multiple-point synchronous implantation and actuation; and the addition of parameter optimization and adaptive algorithms that can further enhance the control effect and biological friendliness.

For swimming, fishes typically rely on the CPG of the brainstem and spinal cord, which is the neural basis of rhythmic swimming movements [104]. Stimulating one side of the midbrain midline of the carp induces opposite turns; forward and backward motions are realized by stimulating the anterior and posterior positions of the midbrain on the midline, respectively [13]. The quantitative control of the steering and forward behaviors can be realized through a combination of different electrical stimulation parameters [14], and the steering success rate is 75% when positive and negative pulses are used [105]. Although the capacity to regulate cyborg fish movements is evident, the absence of a comprehensive fish brain atlas hinders the refinement of stimulation points, reproducibility of control experiments, and control effects. Therefore, further investigation into the quantitative correlations between distinct brain regions and movement patterns is necessary. The remote control of cyborg fish localization is feasible only within a limited range through a global visual localization system, which restricts the application environment and necessitates exploration of breakthroughs in underwater localization technology, remote communication technology, and other domains.

The control of cyborg geckos was achieved using the BCI technique. Turning behaviors were induced by electrically stimulating the MBT brain region, and movement stopped immediately after the stimulation ended [106,107]. Both the intensity and duration of stimulation affected turning angle [19]; therefore, cyborg geckos could walk in a maze by sequentially sending different action instructions [18]. However, the present experiments involved stimulation-induced spinal bending, which led to steering. However, forward, backward, and lateral behaviors are less common and uncontrollable, and the reproducibility of stimulation effects decreases over time because of the unstable fixation of the electrodes to the brain tissues or the impact of multiple stimulations on the nerve [19]. Table 3 compares the control effects of cyborg animals controlled by BCIs.

**Table 3.** Comparison of the performance of cyborg animals controlled by brain–computer interface.

Animal	Stimulated brain regions and parameters				Mobility performance (single stimulation)	Success rate/%	Ref.
Rat	SI MFB	Pw: 0.5 ms Pa: 0.08 mA Pw: - Pa: -	Pf: 100 Hz Dc: - Pf: 0.3–3.0 Hz Dc: -	Pn: 10 Tf: - Pn: - Tf: -	Forward, turn Velocity ~2 m·min^−1^	–	[7]
Rat	SI MFB	Pw: 1 ms Pa: 6~8 V Pw: 1 ms Pa: 10 V	Pf: 100 Hz Dc: - Pf: 100 Hz Dc: -	Pn: 10 Tf: 0.5–1 Hz Pn: 10 Tf: 1–4 Hz	Forward, turn Turning velocity ~3 m·min^−1^	~87.50	[33]
Rat	VPL AMY	Pw: 0.2–0.3 ms Pa: - Pw: 0.2–0.3 ms Pa: -	Pf: 50–100 Hz Dc: - Pf: 50–100 Hz Dc: -	Pn: 5–10 Tf: 0.3–3 Hz Pn: 5–10 Tf: 0.3–3 Hz	Forward, turn	–	[23]
Rat	VPM	Pw: 1.25–12.5 ms Pa: 2–5 V	Pf: 1–100 Hz Dc: 25%	Pn: 15–50 Tf: -	Graded turn Turning angle 60°–120°	100.00	[24]
Rat	NSP	Pw: 0.2 ms Pa: 0.06–0.18 mA	Pf: - Dc: -	Pn: - Tf: -	Graded turn Turning angle ~67°	~41.00	[103]
Pigeon	DIVA	Pw: 15 ms Pa: 5 V	Pf: 250 Hz Dc: 50%	Pn: - Tf: -	Turn	86.00	[39]
Pigeon	DIVA PoA	Pw: 1 ms Pa: 0.3 mA Pw: 1 ms Pa: 0.3 mA	Pf: 25 Hz Dc: 25% Pf: 25 Hz Dc: 25%	Pn: 5 Tf: 5 Hz Pn: 5 Tf: 5 Hz	Forward, turn Turning angle 90°–135° Range 0.1–0.15 m	~75.00	[42]
Pigeon	ICo	Pw: 0.1–0.5 ms Pa: 0.05–0.12 mA	Pf: 50–120 Hz Dc: -	Pn: - Tf: -	Forward	~87.00	[44]
Pigeon	LoC	Pw: - Pa: 1.5 V	Pf: 40–100 Hz Dc: -	Pn: 3–5 Tf: -	Rise, fall Rising distance 12–16 m	87.72–90.52	[48]
Pigeon	FRM	Pw: - Pa: 0.5–1.0 V	Pf: 60–120 Hz Dc: -	Pn: - Tf: -	Turn Turning angle 15°–55° Turning radius 25–135 m Turning velocity 12–15 m·s^−1^	50.00–66.10	[47]
Pigeon	SGP	Pw: 5 ms Pa: 0.1–1.0 mA	Pf: 16 Hz Dc: 10%	Pn: 20 Tf: -	Move, stop	≤66.67	[40]
Gold fish	NFLM	Pw: 3 ms Pa: 0.01–0.1 mA	Pf: 100 Hz Dc: -	Pn: - Tf: -	Forward, turn	–	[13]
Crucian carp	NFLM	Pw: 0–30 ms Pa: 0–10 V	Pf: 0–20 Hz Dc: -	Pn: 50 Tf: -	Forward, turn	~70.00	[105]
Crucian carp	NFLM	Pw: 5 ms Pa: 3–6 V	Pf: 10–100 Hz Dc: 50%	Pn: 40–60 Tf: -	Graded forward and turn Velocity 12–60 m·min^−1^ Turning angle 30°–150°	–	[14]
Gekko gecko	MBT	Pw: 1 ms Pa: 0–0.04 mA	Pf: 50–125 Hz Dc: -	Pn: - Tf: -	Forward, turn	–	[106]

The location of the electrical stimulation, parameters of the stimulation signal, and type of electrical pulse are important factors affecting the success rate in the domain of BCI control [108]. The level of animal movement may depend on the effective charge that flows through the electrodes [109]; a low stimulation frequency does not elicit responses, while high-frequency stimulation can lead to cessation or termination [110]. Despite the efficacy of electrical stimulation in eliciting animal behavior, it cannot regulate the activation of neurons in a spatiotemporally precise manner, which spreads to unintended brain regions that may cause other side effects [111]. In the future, the development of high signal-to-noise ratios and stable electrode arrays, adaptive adjustment of stimulation parameters, tracking, and feedback based on the motion state-space model might improve long-term effective control.

### Electrical Stimulation of Muscles and Receptors

As end-effectors of animal behaviors, muscles contract when nerve cells receive electrical excitation from peripheral inputs such as skin and muscles [112]. Using this mechanism, the complex encoding and decoding process of brain electrical signals can be avoided, and movement patterns can be controlled more directly through different muscle stimulations for animals such as insects with small brain structures and precise movements [113,114]. The most common stimulation signals include constant-current, cathode-guided, and biphasic square-wave signals, among which the biphasic square-wave signal can achieve charge balance because of the alternating phases of the pulses, effectively reducing tissue damage and preventing electrode polarization [115].

With respect to the control of electrical muscle stimulation in cyborg moths, the AL belongs to the central sensory processing node, and the neck muscles (NMs) are directly involved in turning behaviors. Therefore, their low-frequency electrical stimulation can achieve controlled initiation and yaw flight of the cyborg moth, respectively, while high-frequency pulses above 50 Hz immediately stop the flight [54,116,117]. The stimulation of the ventral nerve cord (VNC) can achieve heading control because the abdominal flexion of the VNC can change its flight attitude and then its trajectories [118,119]. Among the three, the stimulation of the AL and NM has a remarkable effect and shares one set of special EMIT probes (EMIT will be especially discussed in the “Electronic Backpack Design” section). Mechanical–muscle coupling is more reliable; however, it can only achieve simple actions with low continuity. The stimulation of the VNC provides multiaction sites and multidirectional adjustable control; however, it suffers from complicated production and operation procedures. Although electrical stimulation can achieve all basic flight actions of moths, most current experiments are tethered experiments, which eliminate the effect of electronic backpack weight. Experiments under free-flight conditions and outdoor wind conditions remain lacking. Therefore, exploring the possibility of achieving continuous multiaction flight control to fully utilize the agility of moth flight while conducting free-flight experiments is essential; however, the primary and important thing is to address the overload control backpack of moths.

**Fig. 4.**
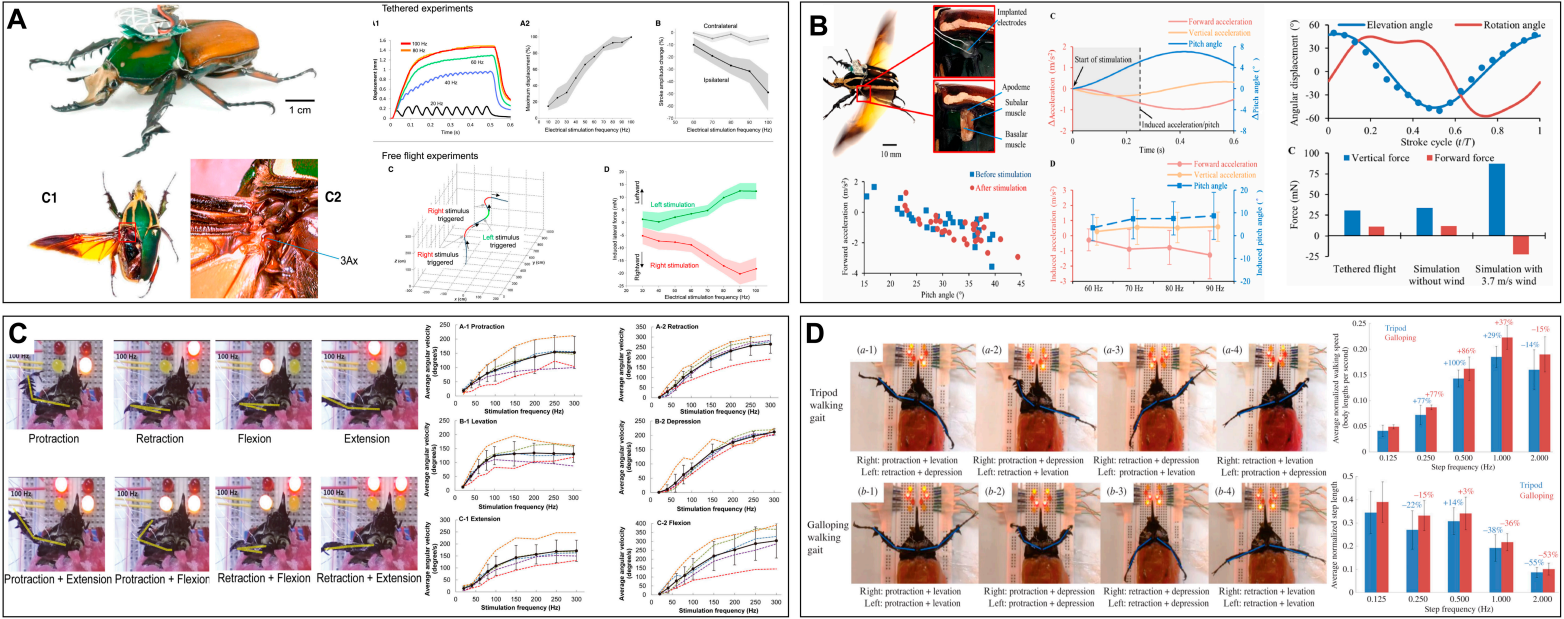
The locomotion of cyborg beetles under the control of electrical muscle stimulation. (A) The same side turning can be induced through the stimulation on the 3Ax muscle of the cyborg beetle. Reproduced with permission [63]. Copyright 2015 Elsevier. (B) Pitching, acceleration, and deceleration can be induced through different stimulation on subalar muscles. Reproduced with permission [62]. Copyright 2022 Elsevier. (C) The 3-dimensional movement of the front legs of beetle can be controlled through closed-loop hierarchical electrical stimulation. Reproduced with permission [70]. Copyright 2014 PLOS. (D) The tripod walking gait and galloping walking gait can be achieved through temporal sequence electrical stimulation of 8 groups of front leg muscles. Reproduced with permission [72]. Copyright 2016 Royal Society.

The basal muscles (BMs) and 3Ax muscles are important muscles of flying beetles that affect flight attitude through wing travel distance and inclination angle. Sato et al. [66] reported that applying positive electrical pulses to the BM triggers a turn to the opposite side. Alternating positive and negative electrical pulses can induce flight initiation and cessation, shortening the pulse interval and affecting the wingbeat frequency, resulting in speed and height control effects [60]. In addition, the 3Ax muscle can induce graded turns to the same side (Fig. 4A) [63]. The stimulation of the BM and 3Ax can increase and decrease the vertical acceleration, respectively, alternating the stimulation of the 2 muscles that can achieve graded control of flight height [69] and turning [67]. Electrical stimulation simultaneously applied to the SMs on both sides causes changes in the flight acceleration and pitch angle of cyborg beetles (Fig. 4B) [62,68]. As beetles crawl through the sequential movements of the 6 legs, the stimulation frequency is positively correlated with the leg joint angle and speed. The closed-loop control system applies the stimulation frequency as a control variable and uses proportional gain and time interval update to adjust the angle error, which can trigger an angle response greater than 12° with a step length error of ≈0.22 cm. The total power consumption of 6 legs is only 5.3 mW (Fig. 4C) [70]. The PWM electrical signals applied to each front LM can precisely control the gait, step frequency, and step length. A tripod gait is produced when the left and right front LMs are alternately stimulated, and a galloping gait is formed when they are stimulated simultaneously (Fig. 4D) [71,72]. The main shortcomings of this method are repetitive errors caused by multiple implants and a lack of freedom in controlled experiments. The suitability of electrodes and muscles and real-drive functional tests remains necessary. When unilateral stimulation is performed on the basis of the touch responses of mechanoreceptors on the insect’s elytra (EL), the lateral walking speed and frequency are directly proportional within the stimulation frequency range of 10 to 50 Hz, and the simultaneous stimulation of both ELs induces a forward speed inversely proportional to the stimulation frequency with a maximum speed of up to 6 cm/s [75]. Although these studies discuss almost all motor patterns of beetles, complex movements and continuous action transitions are difficult and rarely achieved, and the balance of the quantity of inserted electrodes and diversiform motion is an important question that needs to be addressed.

The excellent jumping skill of the locust has the potential to enhance the exploration efficiency of uneven ground compared to beetles and cockroaches and can be divided into 3 stages: flexor muscle (FM) activation, coordinated contraction of the FM and extensor muscle (EM), and flexor release triggering the jump. Under PWM electrical stimulation, the maximum flexion speeds of the flexor and EMs can reach 600°/s and 890°/s, respectively; the simultaneous stimulation of both hind legs can further achieve vertical jumps [120]. Accordingly, Ma et al. [88] designed a bionic timing stimulation strategy: only flexor contraction was induced for the first 200 ms; both muscles were stimulated to contract simultaneously for the middle 200 ms, and only an extensor extension was induced for the last 200 ms, which enables jumps in a fixed direction with a single-leg driving power consumption of only 874 μW. However, this strategy is limited by the low output efficiency of energy conversion from electrical energy to elastic energy and the inability to realize steering jumps; therefore, asynchronous electrical stimulation sequences are used. For example, to induce a right-turn jump, the left hind leg is stimulated earlier than the right hind leg, and the time difference can induce different turning angles [89].

Muscle stimulation offers a more exquisite, powerful, and stable method for cyborg animals; however, it requires more implantable electrodes and power dissipation than the receptor stimulation method. Receptors composed of sensory organs such as the AT, cerci (Cc), and optic lobes (OLs) are important sensory organs for insects. They are connected to sensory nerve endings or the central nervous system with high sensitivity and can direct insects to engage in various activities such as communication, seeking mates, and finding food when stimulated by the external environment [121]. Thus, cyborg insect movement control can be achieved through the electrical stimulation of typical sensory organs.

The AT and cercus are important sensory organs for insects to perceive the outside world; they can encode external touches or air flow directions into reverse turning or moving away motion instructions, and the mechanoreceptor on the AT and loriform hair on the cercus act as core sensors inside [122]. The stimulation of the antenna can cause a brief pitch angle change in the moth and affect the flight height, yaw direction, and speed [57]. The turning behavior of the cockroach in response to antenna stimulation is essentially a reflex triggered by asymmetric sensory input [84]. Therefore, stimulating one antenna would induce the cyborg cockroach to turn to the opposite side, while stimulating both would stop its movement, and the PWM signal to the thorax would make the cyborg cockroach move backward [83]. Besides, alternating electrical stimulation of the left and right AT could achieve line-following movement of the cyborg cockroach [123]. Graded contralateral turns can be induced through the stimulation of the antenna under a constant stimulation amplitude and pulse width and varying pulse frequencies. In addition, graded backward behaviors can be induced by alternating the stimulation of the 2 AT, and behavior freezing appears when simultaneous stimulation is applied. We can consider that the stimulation of the AT is a relatively stable and friendly choice from the responsive maintenance of 1,058 tests within 5 days [73].

**Fig. 5.**
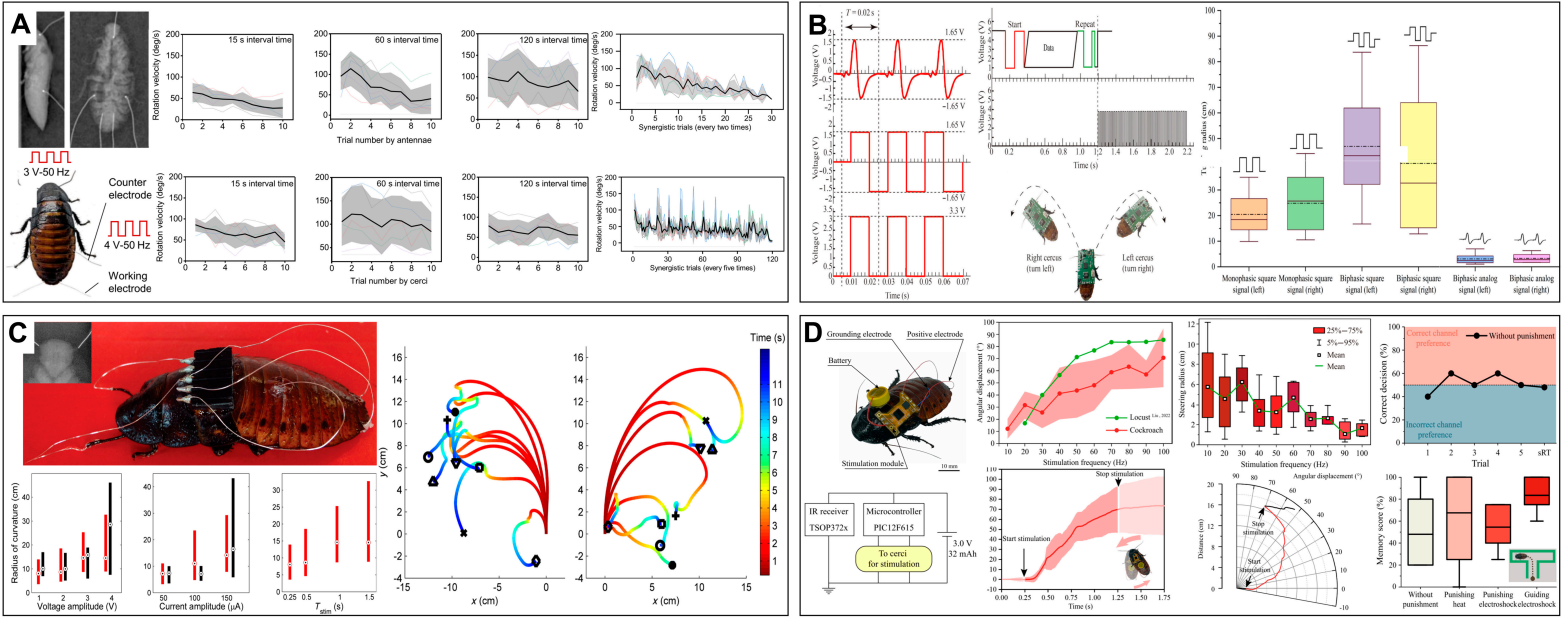
The locomotion of cyborg insects under the control of electrical muscle stimulation. (A) Alternately stimulating the antennae and cerci of cockroach can increase the effective control times to over 100 and reduce the habituation caused by continuous stimulation of a single stimulation pattern. Reproduced with permission [78]. Copyright 2023 AAAS. (B) Applying different stimulation signals to cockroach cerci can trigger different movements of cyborg cockroach; the bipolar analog signal has the best control effect among them. Reproduced with permission [79]. Copyright 2024 AAAS. (C) Different pulse types exhibits different control effects on cyborg cockroach, and the bipolar voltage stimuli are effective for generating sustained and consistent responses. Reproduced with permission [124]. Copyright 2015 PLOS. (D) The turning angle and radius are significantly correlated with the frequency of electrical pulses; the guided electrical stimulation training allows the cockroach to form short-term path preferences. Reproduced with permission [126]. Copyright 2025 AAAS.

The Cc of the cockroach act as emergency escape sensors, where alternating stimulating the AT and Cc can maintain the average rotational speed of the cockroach at ≈45°/s within 100 consecutive stimulation cycles, effectively reducing stimulus habituation and extending the control time (Fig. 5A) [78]. The correlation of the amplitudes of voltage, current pulses, and scales of the induced forward and turning movements can lead to graded control of forward movement and turning (Fig. 5C) [124]. Biphasic analog signals had the best control effect among the different types of electrical stimulation signals and were less likely to cause interface charge accumulation with a stable turning radius, relatively small turning radius, and angular velocity (Fig. 5B) [79]. Applying periodic random stimulation pulse sequences to the AT and Cc enables cyborg cockroaches to perform autonomous exploration in complex environments [125]. Modulated continuous pulses into short burst signals can increase the turning ability by 50% under the same stimulation intensity while maintaining the graded control effect [80]; the effect of guided electrical stimulation training is better than punitive training, and the individuals can form short-term path preferences (Fig. 5D) [126].

Similarly, the electrical stimulation of the Cc of a locust can induce forward jumps with a performance close to that of a natural locust, and the stimulation of the AT can cause contralateral turns; the combined stimulation of both can achieve omnidirectional jumps [90]. Bilateral pulse electrical stimulation applied to the left and right OLs can control the flying of the cyborg bee, and the stimulation over 6 V would stop flight [95], whereas the stimulation of the unilateral OL could lead to a contralateral flying turn [93,94]. Table 4 compares the electrical stimulation parameters of the muscles and sensors and the corresponding movement performances of different cyborg insects.

**Table 4.** Comparison of the performance of cyborg insects controlled by electrical stimulation of muscles and receptors.

Animal	Stimulation site and parameters				Mobility performance (single stimulus)	Success rate /%	Ref.
*Manduca sexta*	AL NM	Pw: - Pa: 3.5 V Pw: - Pa: 3.5 V	Pf: 20–50 Hz Dc: 50% Pf: 20–50 Hz Dc: 50%	Pn: - Pt: - Pn: - Pt: -	Yaw flight, stop Yaw velocity 60.0°·s^−1^ to 80.0 °·s^−1^	–	[116]
*Manduca sexta*	VNC	Pw: 1 ms Pa: 1–10 V	Pf: 50–333 Hz Dc: -	Pn: - Pt: 500 ms	Graded turn	~71	[118]
*Manduca sexta*	AT	Pw: 0.25 ms Pa: 2.8–3 V	Pf: 250 Hz Dc: 50%	Pn: - Pt: 200 ms	Pitch flight Pitch angle ~24.2°	–	[57]
*Mecynorhina torquata*	BM	Pw: 100 Hz Pa: 1.3–3 V	Pf: - Dc: -	Pn: - Pt: 500 ms	Turn, altitude control	~71	[60]
*Mecynorhina torquata*	3AX BM	Pw: - Pa: 1.6 V Pw: - Pa: 1.6 V	Pf: 50–90 Hz Dc: 20% Pf: 90 Hz Dc: 20%	Pn: - Pt: 500 ms Pn: - Pt: 500 ms	Graded altitude control	-	[69]
*Mecynorhina torquata*	3AX SM	Pw: 2 ms Pa: 1.6 V Pw: 2 ms Pa: 1.6 V	Pf: 60–90 Hz Dc: - Pf: 60–90 Hz Dc: -	Pn: - Pt: 500 ms Pn: - Pt: 500 ms	Graded turn	~64	[67]
*Mecynorrhina torquata*	SM	Pw: 3 ms Pa: 3 V	Pf: 40–100 Hz Dc: 10%	Pn: - Pt: 500 ms	Yaw flight, deceleration, stop Deceleration ~150.0 cm·s^−2^ Altitude increase ~200.0 cm·s^−2^ Yaw angle ~20.0 °	–	[68]
*Apis mellifera ligustica*	OL	Pw: 5 ms Pa: 4 V	Pf: 40 Hz Dc: 40%	Pn: 30 Pt: -	Move, stop	≥60	[95]
*Apis mellifera ligustica*	OL	Pw: - Pa: 3 V	Pf: 10–200 Hz Dc: 20–60%	Pn: - Pt: 1,000 ms	Turn	46–90	[94]
*Zophobas morio*	AT	Pw: 2 ms Pa: 2.5 V	Pf: 1–50 Hz Dc: -	Pn: - Pt: 2,000 ms	Forward, graded turn, and backward	≥65	[73]
*Zophobas morio*	AT EL	Pw: 2 ms Pa: 2.5 V Pw: 2 ms Pa: 2.5 V	Pf: 10~40 Hz Dc: 50% Pf: 20 Hz Dc: 50%	Pn: - Pt: 400 ms Pn: - Pt: 200 ms	Forward accelerate, graded turn Turning velocity ≤71.0°·s^−1^	~81	[80]
*Gromphadorhina portentosa*	AT CC	Pw: 500 ms Pa: 0–5 V Pw: 500 ms Pa: 0–5 V	Pf: 50–300 Hz Dc: 50% Pf: 50–300 Hz Dc: 50%	Pn: - Pt: 1.5 s Pn: - Pt: 1.5 s	Graded forward and turn Range 15.6–25.6 cm Velocity accelerate 3.9–5.6 cm·s^−1^ Turning angle 80.0°–149.0° Turning angular velocity 41.2–62.8°·s^−1^	41–56	[124]
*Gromphadorhina portentosa*	CC AT	Pw: 1,000 ms Pa: 4 V Pw: 1,000 ms Pa: 4 V	Pf: 10–100 Dc: 50% Pf: 10–100 Dc: 50%	Pn: - Pt: - Pn: - Pt: -	Turn, acceleration Turning angle ~38.5° Velocity accelerate 60.0 mm·s^−1^ Turning angular velocity 60.0°·s^−1^	~74	[156]
*Locusta migratoria*	EM FM	Pw: 2 ms Pa: 2 V Pw: 2 ms Pa: 2 V	Pf: 30–200 Hz Dc: - Pf: 30–200 Hz Dc: -	Pn: - Pt: - Pn: - Pt: -	Graded forward jump Jump range ~42.6 cm Jump altitude ~10.4 cm	–	[88]
*Locusta migratoria*	EM FM	Pw: 2 ms Pa: 2 V Pw: 2 ms Pa: 2 V	Pf: 80 Hz Dc: - Pf: 100 Hz Dc: -	Pn: - Pt: - Pn: - Pt: -	Forward jump, turning jump Jump range ~40.0 cm Jump turning angle ~20.0°	–	[89]
*Locusta migratoria*	AT CC	Pw: 2 ms Pa: 3.2 V Pw: 2 ms Pa: 3.2 V	Pf: 20–60 Hz Dc: 50% Pf: 20–60 Hz Dc: 50%	Pn: - Pt: 1,000 ms Pn: - Pt: 1,000 ms	Forward jump, turning jump Jump range 12.3–33.4 cm Jump altitude 5.6–9.4 cm	~60	[90]

The similarity between muscle stimulation control and receptor stimulation control includes considering charge balance and stable interface impedance, reasonable selection of parameters of the stimulating current, and adaptation and fatigue caused by prolonged stimulation. Meanwhile, there are also differences; for example, muscle stimulation acts on the end-effector and directly causes action changes, while receptor stimulation induces behaviors through bogus feelings, and muscle stimulation requires a higher current density and has low latency. We believe that low impedance, high signal-to-noise ratio, high-stability electrode configuration, adaptive stimulation regulation, anti-fatigue strategy of multimuscle group alternating stimulation, and bionic signal encoding based on a natural stimulation structure can assist in refining this control mode.

### Visual stimulation

The control of visual stimulation utilizes the instinctive behavior of animals, wherein visual effects within the animal can positively or negatively induce them to move in a specified direction.

Black barriers at different positions within the vision of the turtle can make it move in the opposite direction because of its good perception and memory; therefore, the turtle can be guided along a predetermined path to achieve forward movement, stopping, and turning by alternately applying no stimulation and obstacle avoidance stimulation [20]. Multiple light-emitting diodes (LEDs) were installed at intervals within a 120° field of view in front of the turtle. When the turtle effectively responds to LED stimulation, the gel-type food is fed after the relationship between the target waypoint and the current position of the turtle is calculated. The LED can be controlled to guide the turtle to pass through the waypoints in sequence. The average trajectory deviation error of the turtle is 18.83 cm after animal training [22]. Although this method has a small effect on animals, the stability and accuracy of the system may be affected when similar light sources, predators, or prey appear simultaneously in the field of vision, and the scale changes of states are difficult to maintain consistently. Peng et al. [15] controlled the cyborg carp with red and blue LED lights and reported that the control effect was related to both the position of the light source relative to the position of the eyes of the fish and the color of the light source. The average control success rates of the red and blue lights were 73.7% and 67.8%, respectively. For underwater light stimulation control, fluctuations in the external light intensity, optical and electrical characteristics of the light source, and environmental noise may have effects. Moths [55] and beetles [65] performed yaw flights following the rotational direction of a pattern on a circular LED array. The LED array is set to create a light flow image moving away from both sides of the bumblebee; the bumblebee flies forward and follows the yaw if the light flow rotates unidirectionally [127].

Consequently, the development of portable light stimulation devices that are highly resistant to interference and have stable light source characteristics and long battery endurance is essential. Examples of such devices include light shading and concentrating structures, narrowband LEDs, and those with adaptive filtering algorithms.

### Other stimulation methods

In addition to the aforementioned control strategies, there are many other stimulation control techniques for individual cyborg animals that exploit their special sensory responses or predatory characteristics.

#### Simulated predatory electric field stimulation

Kajiura and Fitzgerald [11] controlled a shark by manipulating the spacing of the dipole and current intensity between the electrodes. Sharks bite the electric fields created by the activated electrodes under the guidance of their hunting instincts. The greater the electrode spacing and current intensity, the farther away the cyborg shark can orient from it, with the maximum response distance reaching 46.1 cm. However, this method is susceptible to changes in the geomagnetic field, and its anti-interference ability and wide application are limited and can have certain species-specific characteristics. Consequently, it may be worthwhile to further verify the absolute sensitivity of sharks to electric fields and develop a relatively stable control strategy.

#### Chemical substance stimulation

Artificially injected chemical substances exert a specific influence on the neurotransmitter system, affecting the normal decision-making of the motor control network of the brain and eventually leading to observable and directional changes in the intensity, pattern, behavior, and so on.

Cyborg moths can be rapidly induced into a chemically paralyzed state (from complete flight activity to no movement) within 90 s by injecting the solution through a microfluidic chip containing saturated L-glutamic acid or L-aspartic acid solution to the dorsolongitudinal flight muscles of the thorax and can fully recover within 22 h [58]. Further, flight output power regulation can be combined with electrical stimulation flight control. Compared with mechanical stimulation, this can increase the continuous flight time by 35 times and reduce the average flight power output by 50% [59]. Lin et al. [87] reported that the moving range could be increased by up to 342% when saturated methyl salicylate was applied to the hind legs of cockroaches. The range of the activity speed could also be increased; however, each stimulation lasted for 20 to 25 min without affecting the response to the electrical control. The terrain coverage velocity of the dual-modal control method combined with random electrical stimulation was 21.6 cm^2^/s, which was significantly better than that of the single control strategies. Compared to free insects, the movement speed increased by 24.3%, and the coverage efficiency increased by 11.9 times.

Long-term drug resistance and toxicity testing are indispensable processes for chemical substance stimulation control. Subsequently, precise control and autonomous quantitative drug release triggered by action states can be achieved by adjusting the application method and application site of a drug while reducing the impact on health. The ultimate strategy for maximizing and optimizing drug incentives for cyborg animals with alternating multistimulation strategy coordination and regulation through closed-loop sensing can be established.

Table 5 provides a comparison about the functioning mechanism of different chemical substance stimulations.

**Table 5.** Comparison of the cyborg animal influences under different chemical substance stimulations

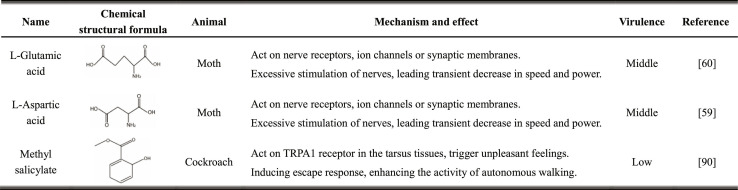

#### Thermal stimulation

In terms of influencing animal behavior, thermal stimulation employs 2 effect mechanisms: a feeling cue and physiological accommodation. For example, the beetle deflects at ≈15° [52] when the interface temperature between the beetle and the heater reaches 43 °C. In addition, only 330 to 360 mW is required to achieve an average deflection angle of 30° to 45° with a success rate of 93.5% through the piezoelectric heater at the resonant frequency [53]; these were achieved based on “thermal directed escape”. In contrast, an example of physiological accommodation includes 808 nm light that targets the noninvasive “plasmonic nanotattoo” composed of gold nanorods and ultrathin silk fibroin interfacial films on locust wings. This can increase the wing temperature by 20 °C within 20 s and cause the cyborg locust to flee [91]. Thermal stimulation can decrease the preheating time of beetles and cockroaches before takeoff from 5 to 10 min to only 56 s [56]. In addition, thermal stimulation can affect the population behavior and distribution of insects. Barmak et al. [128] utilized an intelligent beehive equipped with an array of thermal sensors and heaters to adjust the spatial distribution of bee colonies based on thermal stimulation. This system can autonomously intervene in the behavior of the bee colony in a closed-loop mode, enabling the group to move up to 9.3 cm per day.

Low-temperature thermal stimulation is a noninvasive method that addresses the dependence of traditional neural electrical stimulation on specific physiological structures by leveraging the natural heat avoidance behavior of insects, thereby providing a more universal and less damaging solution for cyborg animals.

#### Light stimulation

**Fig. 6.**
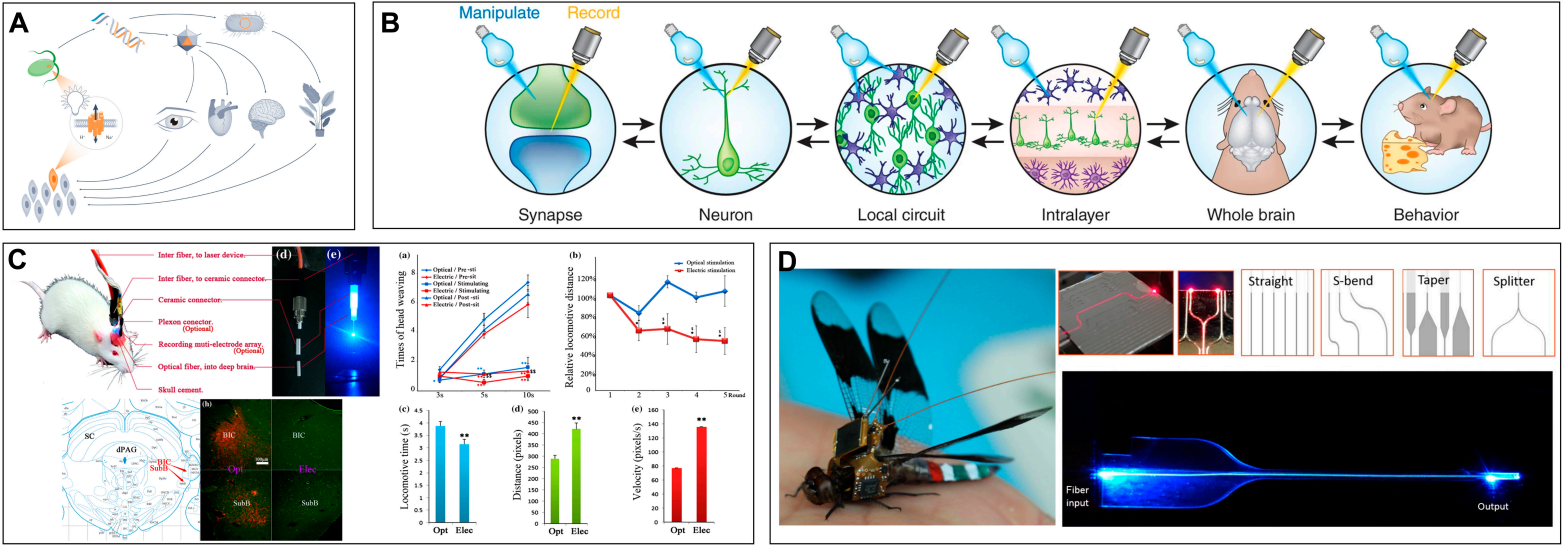
The principle and achievement of light stimulation based on optogenetics. (A) Principles of optogenetics that show the DNA resources of the sensory photoreceptor, packing and injection, and light control. Reproduced with permission [129]. Copyright 2022 Springer Nature. (B) The use of optogenetics at all levels of brain function. Reproduced with permission [130]. Copyright 2014 Springer Nature. (C) The cyborg rat can be controlled under electrical stimulation and light stimulation; the optical stimulation affected only dPAG and has higher accuracy. Reproduced with permission [26]. Copyright 2015 Springer Nature. (D) Submillimeter-level control of dragonfly can be achieved under light stimulation. Reproduced with permission [96]. Copyright 2017 SPIE.

Optogenetics, which combines optics and genetics, forms the core of light stimulation. The general procedure involves infecting cells with appropriate photosensitive proteins using viral vectors and then using an optical fiber laser to express the light-sensitive channels in the target cells to precisely control specific types of neurons in the brains, spinal cords, or peripheral nerves (Fig. 6A) [129].

The light stimulation of the dorsal periaqueductal gray (dPAG) brain region of rats can selectively activate the dPAG and downstream circuits, encoding freezing and escape behaviors with stable control effects (Fig. 6C) [26]. A flexible optical waveguide-based optogenetic system with multichannel waveguide arrays can precisely activate target-selective directional neurons in the dragonfly nerve cord at a wavelength of 473 nm and achieve submillimeter-level control by decoding the flight direction (Fig. 6D) [96].

In contrast to direct muscle stimulation, light stimulation shows high spatial and temporal accuracy, target specificity, and extremely low diffusivity because the “key” of light stimulation (specific wavelengths of light) can only open cells that have a specific “lock” (light-sensitive protein) [26]. In addition, compared with visual stimulation, its top-down control affects fewer neural circuits and has a short influence range. However, the high invasiveness, complex and cumbersome operational techniques, influence of viruses and fiber optics, and low interpretability of physiological reactions must be addressed [130].

### Technical comparison

Different control methods have different applicability, advantages, and limitations; therefore, choosing the most suitable method to achieve different goals is crucial, as are the comparisons and discussions of the 7 control techniques mentioned above.

In terms of animal adaptability, BCI-based and optogenetics-based light control are applicable to animals with relatively high intelligence, complex regulation of motor skills, and a wide variety of movement patterns. The other methods have no clear tendency for animal selection; however, the existing methods are quite different. For example, the predatory electric field control method is only applicable to sharks, which indicates species specificity. For such instances, zoological research is necessary. Biocompatibility depends on whether there are invasive surgeries, the properties of the materials in direct contact with tissues, and whether the control method causes damage to the animals. Among these methods, noninvasive control methods such as vision-based and electric field-based methods have higher biocompatibility. The effect of heat-based methods is uncertain because studies on the effects of local low temperatures on the bodies of various animals are relatively scarce. Further, chemical substance stimulation is a good control method if the decomposition of a chemical substance is rapid and the side effects are minor. As craniotomy and material implantation are indispensable in BCI- and optogenetics-based control methods, the vulnerability of the brain and long-term stability of materials make it difficult to finish the surgery and maintain the stability and fineness of the brain tissue–material interface; therefore, the concern for animal health and the effectiveness of control measures are of great importance. For behavior control accuracy, the stimulation of muscles and optogenetics-based methods show higher accuracy on both temporal and spatial dimensions, and closed-loop control can be easily established because the brain is the core of multiaction regulation. Vision-based and electric field-based methods show lower accuracy, which can be attributed to the susceptibility to environmental interference and imprecise instructions. Furthermore, the existence of the onset time of drugs makes the lowest timeliness of the chemical substance stimulation. Owing to the construction difficulty of cyborg animals, from designing their movement to roboticization, BCI-based cyborg animals are the most complex, followed by the muscle-receptor methods, heat-based methods, optogenetics, and electric field stimulation, with the drug-based and vision-based methods being relatively easy.

## Electronic Backpack Design

An electronic backpack is a general term for integrated electronic devices that are attached, worn, or fixed to the body of a cyborg animal. The electronic backpack is responsible for receiving commands from the upper computer, converting the commands into stimulation instructions or instruction sequences, directly controlling the movement of the cyborg animals through electrodes or other noninvasive devices, and measuring and reversing the transmitting state parameters. These include microelectrodes, microcontrollers, batteries, sensors, carrying (or fixing) parts, solar panels, sealed shells, and other supporting devices.

### Overall design

Although different animals have different body sizes, weight-bearing capacities, and control strategies, all electronic backpacks have relatively consistent design criteria, namely, small size, low weight, low power consumption, and high reliability.

#### Electronic backpacks for terrestrial cyborg animals

**Fig. 7.**
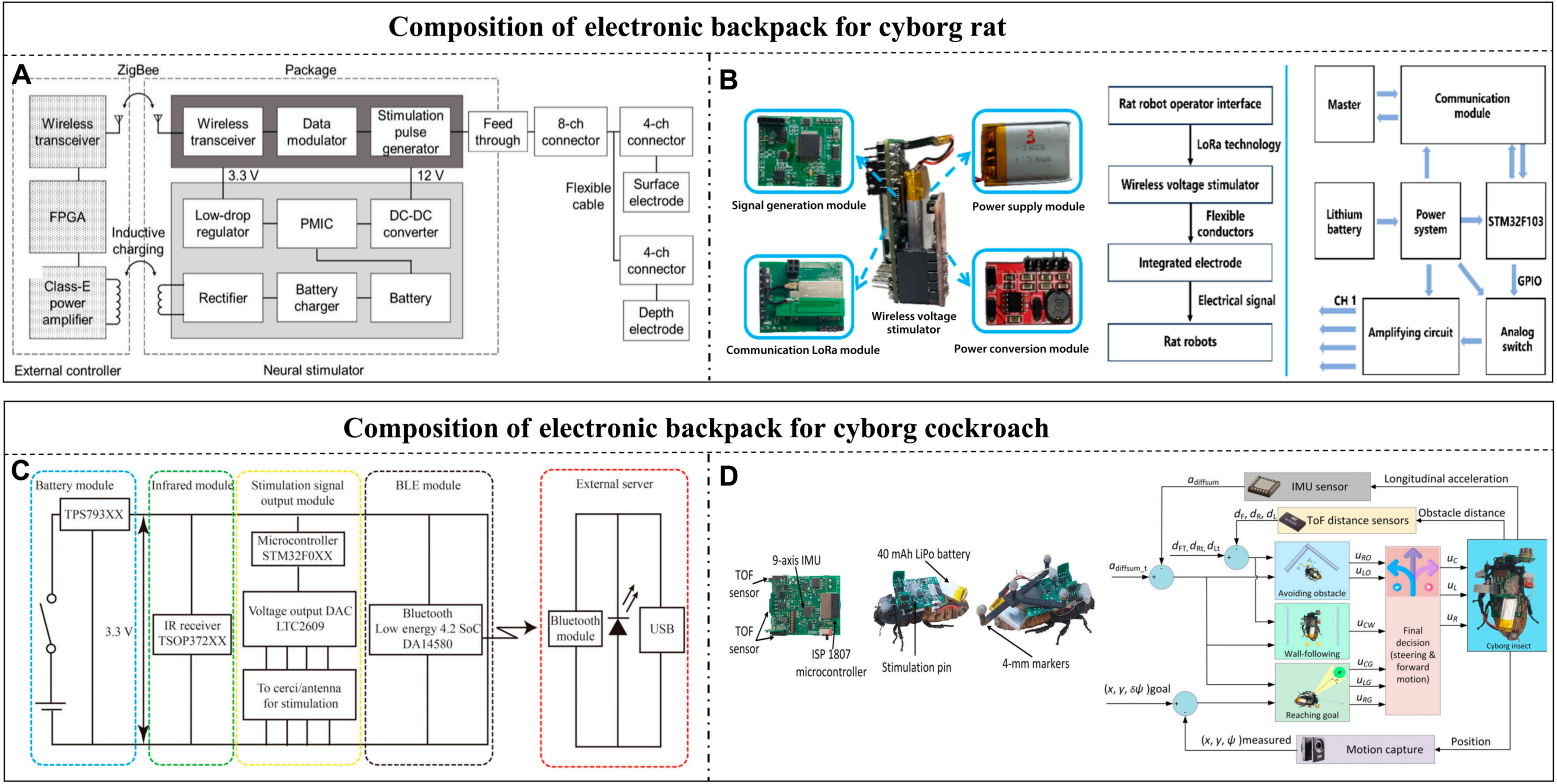
Electronic backpacks of terrestrial cyborg animals. (A) Electronic backpack block diagram of the cyborg rat that controlled 2 stimulating electrode arrays. Reproduced with permission [25]. Copyright 2017 SPIE. (B) The framework of the neural stimulation system and the wireless voltage stimulator for the cyborg rat based on LoRa. Reproduced with permission [132]. Copyright 2019 MDPI. (C) The schematic diagram of the cyborg cockroach locomotion control system with both IR controller and Bluetooth-based upper computer. Reproduced with permission [79]. Copyright 2024 AAAS. (D) The electronic backpack with the 9 DOF IMU and TOF sensor can be used to navigate the cyborg cockroach under an external 3D motion capture system. Reproduced with permission [136]. Copyright 2025 Mary Ann Liebert, Inc.

Terrestrial animals have the fewest constraints in electronic backpack design because of their relatively stable movement state. Electronic backpacks for cyborg rats use C8051 [131], ESP32 [84], or STM32 [132] as the main controller, which is directly connected to the stimulation electrodes outside the body with flexible wires and communicates with the PC or handset in real time via FM [133], Bluetooth [34], ZigBee (Fig. 7A) [25], and LoRa (Fig. 7B) [132]. The components are surface-mounted devices to reduce weight and size, and the backpack is fixed to the backpack with Velcro [131]. The total weight, including the battery, is usually 5 to 20 g. Researchers test the control and navigation performance of cyborg rats in a maze, and IMU modules such as BMI160 [34] and MTi-3 [30] are added to the backpack to obtain real-time state parameters and position data. Precise navigation can be achieved with overhead cameras. To address the issues of wound infection and damage caused by implanted stimulators from external force collisions, researchers developed a magnetic coupling-implanted stimulator encapsulated in a silicone material that can achieve the full-angle coverage control of rat movement under the drive of a pulsed magnetic field [134]. In addition, multilayer encapsulation composed of a parylene conformal coating and silicone has been used for enhancing waterproofing performance, enabling continuous operation in the body for approximately 5 months [135]. Seo et al. [45] developed a fully implanted stimulator encapsulated in a liquid crystal polyester with lower moisture absorption; the microcontroller was made of a flexible PCB, which provides excellent control performance and biocompatibility. The effective load capacity of beetles and cockroaches is ≈3 g; therefore, microcontrollers such as SAMD21 [85] with higher integration are more favored, while ZigBee [123], low-energy Bluetooth (Fig. 7C) [79], and WiFi [78] are used for communication. The total weight of the electronic backpack, including the battery, is ≈1.3 g, and it is ≈6 g when equipped with a camera, IMU, or other modules [85]. In 2025, Ariyanto et al. [136] reduced the weight of the backpack equipped with an IMU, camera, and laser ranging module to 2.9 g, achieving better functionality and a lower burden for a cyborg cockroach (Fig. 7D). Further, the backpacks of cyborg insects are adhered directly to the backs of insects using beeswax or hot melt.

Generally, the backpacks of large cyborg animals such as rats preferably utilize commercial-grade hardware modules, while small cyborg animals use simplified or customized elements that have low integration and miniaturization. With the development of integrated circuits, smaller controllers with higher functional integration, such as ESP32S3, are gradually adopted. Some of them even incorporate certain artificial intelligence (AI) functions. The efficiency rate of development and the intelligence level of electronic backpacks are expected to increase.

#### Electronic backpacks for aerial cyborg animals

**Fig. 8.**
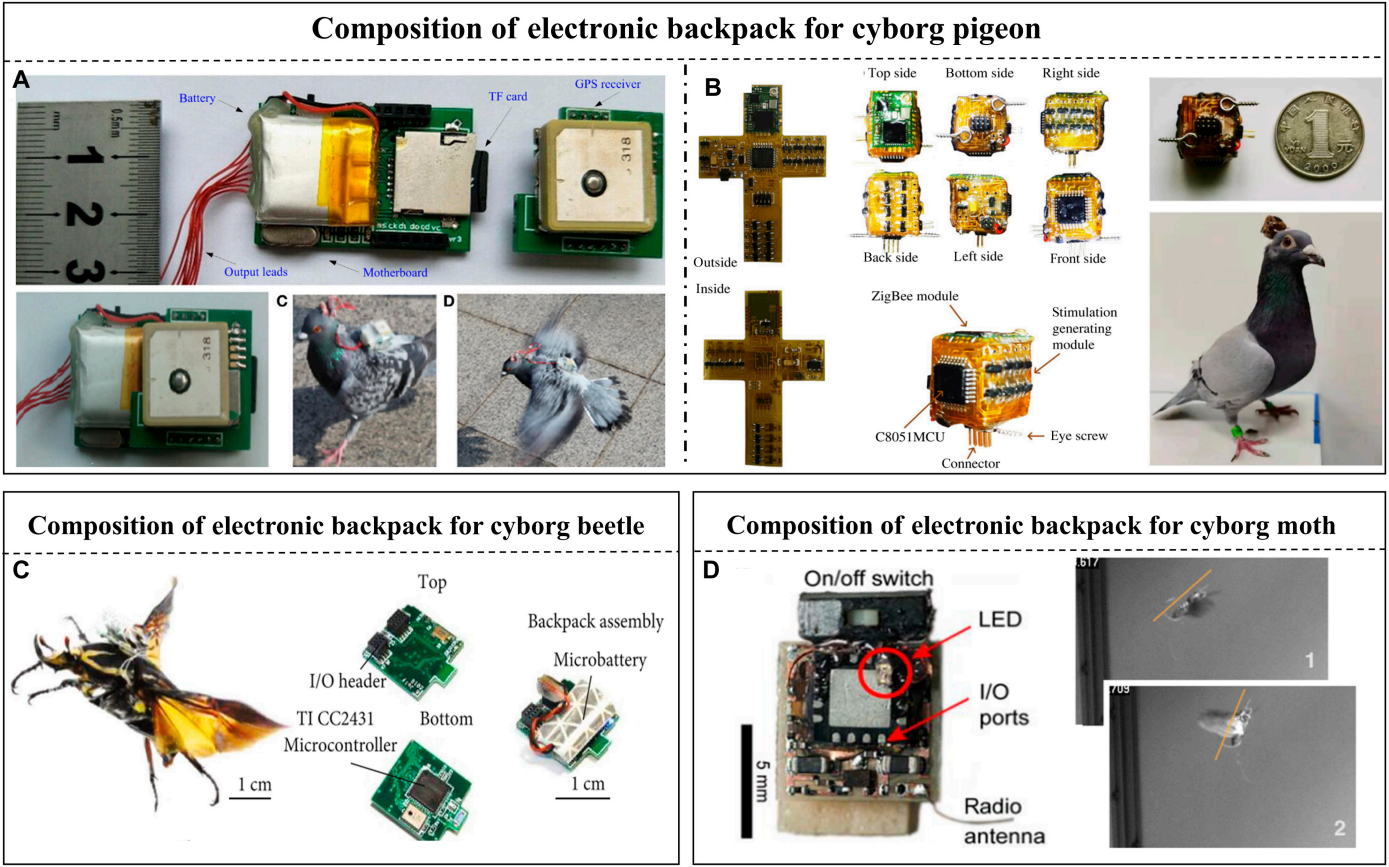
Electronic backpacks of aerial cyborg animals. (A) The electronic backpack with a micro-GPS module and TF card can be used to monitor and control the cyborg pigeon to soar outdoors. Reproduced with permission [49]. Copyright 2017 Frontiers. (B) The adoption of the flexible PCB and cube structure makes the electronic backpack lighter and smaller for better application to flight pigeon controlling. Reproduced with permission [140]. Copyright 2023 Royal Society. (C) Simplified electronic backpack with retro-reflective tape can control the flight of the cyborg beetle and be monitored by the camera system at the same time. Reproduced with permission [68]. Copyright 2022 AAAS. (D) The electronic backpack with a weight of only 0.42 g can be mounted on the ventral side to control the turning behaviors of the cyborg moth. Reproduced with permission [57]. Copyright 2012 PLOS.

Electronic backpacks for aerial cyborg animals prioritize weight and communication distance. The basic components include a microcontrol unit, a communication module, and a stimulation module. The electronic backpacks of cyborg pigeons contain a positioning module for outdoor flights (Fig. 8A) [49], which weighs more than 15 g. Communication methods include general packet radio service (GPRS) [137], ZigBee [138], and radio frequency (RF) [139]. The GPRS module can achieve long-distance and high-transmission-rate communication with a control range of up to 3 km outdoors, while ZigBee and RF consume less power in short-range communication. The cubic stimulator developed by Liu et al. [140] exploits the high wiring density, light weight, and thinness of flexible PCBs to significantly reduce the weight of the electronic backpack to 4 g and reduce the volume from an average of 20 to 3.375 cm^3^ (Fig. 8B). The communication distance is ≈120 m in indoor environments and can reach up to 150 m in outdoor environments.

The electronic backpacks of flying cyborg beetles also include IMU sensors to detect flight attitude (Fig. 8C) [68]. The electrical stimulation pulse train is mapped using custom signal generation software from the flight command and applied to cyborg insects to induce movement [62]. Further, a 3D motion capture system can be used to track reflective markers on backpacks to coordinate and synchronize the insects using control software to achieve real-time positioning and flight control [69]. For small flying insects such as moths, which only have a load capability of ≈1 g, microcontrollers such as Atmel Tiny [51], PIC12 [54], and PIC16 (Fig. 8D) [57] are used, and the total mass of the backpack is ≈0.5 g. Moreover, a balloon is used for weight balancing to reduce the load impact. The transmission distance is ≈50 m, and it can work continuously for more than 5 h [116,117]. However, this type of flight backpack, which requires balloon assistance, is unsuitable for complex environments because balloon traction can affect the flight of the moth.

For backpack matching and flight endurance, the primary constraint is the weight of the backpack; however, Li-Po or other solid-state batteries account for more than half of the total weight. Simultaneously, the weight and energy density affect the reserve of the electricity of batteries; therefore, the balance between endurance and battery weight remains a challenge. Novel techniques such as bioenergy harvesting [141] and piezoelectric energy harvesting based on flapping [142,143] can assist in elevating endurance and reducing pressure during flight.

#### Electronic backpacks for aquatic cyborg animals

Underwater wireless communication and sealing are the key features of electronic backpacks for aquatic animals. A 900-MHz radio communication [144] and 433-MHz RF communication [145] are adopted, while low-density materials such as polystyrene are added to electronic backpacks to offset the weight of the backpack and reduce its impact on the fish swimming [13,146]. Although the buoyancy of the low-density material balances the weight of the backpacks, the volume is still relatively large, and the shape and material do not fit well with the fish, affecting the normal swimming posture. Meanwhile, the waterproof material of the backpack is silicone resin, which is not sufficiently stable or safe for fish [13]. To address these problems, Hou [145] designed a backpack that suits the shape of the carp, and the sealed battery and circuit board were placed on both sides to maintain balance and ensure natural swimming. For aquatic cyborg animals, ocean exploration and deep-water exploration are important working scenarios to leverage the underwater advantages of biological organisms, and there are high requirements for long-distance underwater communication and stable and highly compatible sealing techniques under high-water-depth, high-pressure, and no-light conditions. Techniques such as minisonar [147] and visible light communication [148] can provide new ideas for subsurface communication, and the use of superhydrophobic coatings [149], composites for anticorrosion [150], underwater adhesives [151], and underwater self-healing piezo-ionic elastomers [152] can enhance the subsurface waterproofing, anticorrosion, sealing, and self-repairing performance, respectively.

These different requirements lead to different plans, and Table 6 summarizes the design and performance of several typical electronic backpacks. The choice of the communication mode varies according to different working conditions. The low-rank adaptation is the most adaptive technique for different types of environments, such as indoors or parks. In particular, its indoor coverage performance, power consumption control, and anti-interference capability are excellent. However, it works at a low transmission rate, which is not suitable for video and high-speed transmission. The GPRS can be considered optimal in remote control missions to achieve wide-area network coverage with bidirectional communication; however, data management and cost control should be noted. In addition, acoustic communication shows lower signal attenuation than RF and better communication distance underwater; however, the high latency characteristic of sound speed and complex noise treatment cause some difficulties.

**Table 6.** Comparison of the electronic backpacks of different species

Species	Controller	Communication mode	Control distance /m	Size/mm	Endurance /h	Weight /g	Ref.
Rat	C8051F330	FM	200	25 × 15 × 2	1	10.0	[131]
Rat	STM32F103	LoRa	3,000	–	–	–	[132]
Rat	–	Zigbee	400	29 × 26 × 8	13.0	5.9	[25]
Rat	STM32F407	Bluetooth	–	36 × 38 × 26	8.0	9.1	[34]
Pigeon	ATmega8L	–	–	38 × 26 × 8	2.3	18.0	[49]
Pigeon	C8051F006	RF	–	26 × 16 × 9	–	9.0	[213]
Pigeon	STM32F103	GPRS	–	34 × 24 × 20	–	19.0	[137]
Pigeon	STM32F103	RF	100	30 × 16 × 1.5	–	15.8	[139]
Pigeon	C8051F410	ZigBee	150	16 × 18 × 16	6.0	4.0	[140]
Carp	STM32L151	RF	–	–	–	–	[145]
Beetle	CC2431	RF	–	–	–	1.3	[62]
Beetle	CC2530	RF	–	15 × 15	–	1.3	[67]
Cockroach	STM32H743	WiFi	15	–	–	5.5	[78]
Cockroach	STM32F051	IR+Bluetooth	–	20 × 35	2.0	6.2	[79]
Cockroach	ESP32-CAM	Bluetooth	–	–	–	–	[84]
Cockroach	SAMD21G18	RF	–	–	0.8	6.0	[85]
Cockroach	PIC16F630	ZigBee	–	–	–	4.0	[123]
Cockroach	ISP1807	Bluetooth	–	–	–	2.9	[136]
Moth	PIC12F615	AM	–	–	5.0	0.65	[54]
Moth	PIC16F688	AM	–	6.8 × 10.2 × 5.1	–	0.42	[57]
Moth	Atmel Tiny13V	–	–	8 × 7 × 1.5	–	0.5	[51]

In addition, in the design of electronic backpacks, low-power and highly integrated controllers that support basic waveforms such as square and sine waves, meet the precision requirements of the DAC resolution and PWM frequency, and have multichannel output functions are typically selected. In addition, gain amplifiers are added to the circuit to enhance weak signals, expand the dynamic range, match the driving capability, manage the signal-to-noise ratio to ensure signal integrity, and improve the performance of electronic backpacks.

### Design and implantation of microelectrodes

From the perspective of the control effect and long-term operation of cyborg animals, the electrode implantation is a decisive factor. The material types, implantation sites, implantation methods, and sealing and fixation methods all require rigorous designs.

**Fig. 9.**
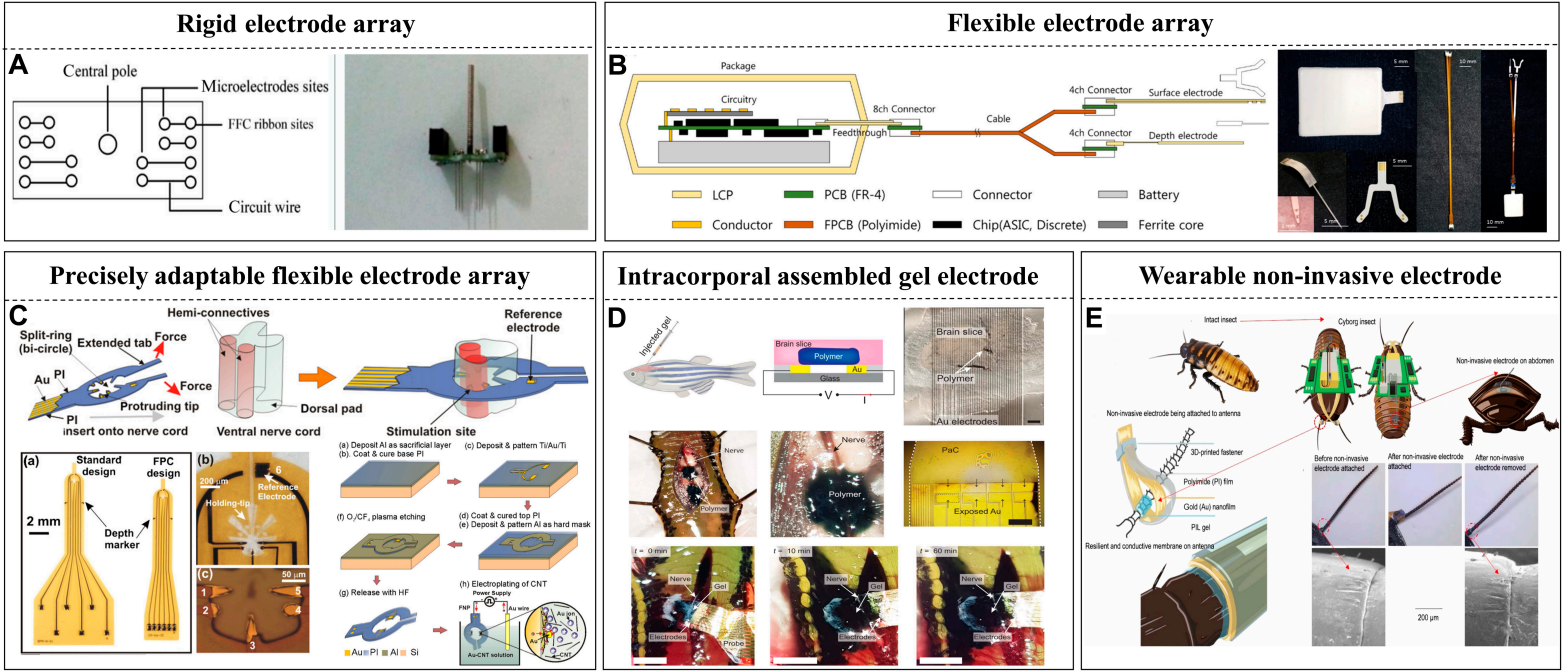
Different microelectrodes for different cyborg animals. (A) The multiple brain regions’ synchronization implanted microelectrodes for pigeon. Reproduced with permission [37]. Copyright 2016 Taylor & Francis. (B) The compositive structure of the depth-type electrode array and surface-type electrode array for rat. Reproduced with permission [25]. Copyright 2019 MDPI. (C) The design and manufacture of the circular flexible neural probes for moth. Reproduced with permission [119]. Copyright 2012 Elsevier. (D) Using endogenous metabolites to trigger the enzymatic polymerization of organic precursors within an injectable gel can form conducting polymer gels as electrodes in vivo. Reproduced with permission [155]. Copyright 2023 AAAS. (E) The conformal electrodes with a resilient and conductive membrane used for noninvasive mounting on cockroach antennae. Reproduced with permission [156]. Copyright 2023 Springer Nature.

Implantable microelectrodes are made of stainless-steel wires with polytetrafluoroethylene coatings [23], insulated nickel–chromium alloy wires [47,103], tungsten wires [153], or platinum wires [146], which are often used to improve the transmission frequency and anti-interference ability. The length and diameter of the electrodes vary based on the size of the animal, location of the brain region, and stimulation accuracy. Electrodes with submillimeter diameters are the most widely used because of their stable and accurate characteristics. In traditional methods, each pair of electrodes corresponds to only one brain region, which is not conducive to multisite stimulation and convenient surgical implantation. Consequently, multiregional synchronous implantable microelectrodes (Fig. 9A) [37] and high-integration multichannel electrode arrays [154] are effective. Tsang et al. [119] developed a flexible ring-shaped electrode array with 9 stimulation sites that significantly reduce the interface capacitance, impedance, and charge density and improve the signal-to-noise ratio (Fig. 9C). To address the incompatibility between traditional rigid electrodes and living tissues and the risk of tissue damage, Strakosas et al. [155] developed a soft, baseless conductive polymer gel electrode directly formed in the brain, fins, and heart of zebrafish that realizes in situ polymerization through endogenous metabolites and maintains stability in complex biological environments (Fig. 9D). Lin et al. [156] developed a noninvasive conformal electrode composed of polyionic liquid gels and gold nanofilms; the polyionic liquid gel was used to achieve complete contact between the electronic conductor and the body of the insect and conduct electrical stimulation, successfully achieving stimulation navigation with no obvious damage on AT (Fig. 9E). In addition, a fully implantable wireless stimulator encapsulated in a liquid crystal polymer (LCP), which matches the curvature of the body of the animal, has high stability and reliability and is conducive to reducing the difficulty of surgery (Fig. 9B) [25]. However, few studies have focused on and analyzed the changes in implanted electrodes during the long-term experimental process but only discussed animal responses and mission finish rate after several stimulations. Certainly, changes after electrode insertion after the experimental period are not considered. Su et al. [38] mentioned that most experimental pigeons show a gradual decline in sensitivity to electrical stimulation around 20 days after the surgery, and their responses to the stimulation disappeared around 40 days after the surgery. A very small number of pigeons can maintain sensitivity to the stimulation unchanged; however, the dental cement used for fixing electrodes failed within 2 months, and the pigeons survived with no controlled behaviors. In my opinion, monitoring and tracking the complete experimental period of cyborg animals is necessary, which not only encourages researchers to optimize and iterate experiments to create more stable and reliable cyborg animals but also makes it convenient to take care of the health and welfare of animals.

All microelectrode implantation surgeries are standardized in accordance with the “Guide for the Care and Use of Laboratory Animals” (National Academy of Sciences, USA), “Regulations for the Administration of Affairs Concerning Experimental Animals” (State Scientific and Technological Commission, CN), and other regulatory standards. The first step of surgery is anesthesia; rats are anesthetized by injecting chloral hydrate [103], ketamine and xylazine [26], or pentobarbital sodium [27]; pigeons are anesthetized by combining pentobarbital (general anesthesia) with lidocaine hydrochloride (local anesthesia) [42], chloral hydrate [44], or inhaled isoflurane [45]; fish are anesthetized by clove oil solution [105] or MS-222 water solution [144]; and insects are anesthetized by carbon dioxide [67] or cold treatment (4 °C) [123]. After successful anesthesia, the heads of the animals were placed in a stereotactic instrument for fixation, the implantation sites were exposed, and drilling and electrode implantation were performed. With the exception of insects, which were punctured using insect pins, a cranial drill is used to drill the sites of the larger animals. The insulating layer at the tip is partially removed, and the electrodes are implanted based on the positioning reference. Electrodes for rat brains are fixed with stainless steel screws and dental cement [26], and the electrodes for insects are fixed with materials such as beeswax [67,79], while the gaps between the electrode and brain tissue of pigeons and fish are filled, reinforced, and sealed with dental cement [14,105], hot melt adhesive [157], or EC glue [43]. However, these sealing materials and methods have poor biocompatibility and may have insufficient sealing performance, especially after craniotomy, which makes it difficult to ensure the long-term survival of animals. Therefore, developing degradable temporary implantable electrode arrays, implantable electrodes with tissue healing-promoting material coatings, nonimplantable electrodes, or minimally invasive electrodes can help improve the success rate of control and biocompatibility. Further, it is necessary to ensure a good seal between the electrode and tissue, increasing the amount of hydrogen bubbles generated during electrolysis and easily causing trauma to the muscles of aquatic animals [99].

Early metamorphosis insertion electrode implantation technology for cyborg moths is unique and effective. Approximately 7 days before pupation, the micro-nano-fabricated flexible electrodes are implanted into the muscle tissue through an incision in the epidermis. Cyborg moths with electrodes implanted during the pupal stage heal the surrounding tissues and form a permanent fixed structure after they grow into adults. The successful emergence rate can reach ≈90% [158], and the electrode–tissue interface impedance is considerably lower than that of the electrodes implanted in the adult state, whereas the charge storage capacity increases [159]. This special electrode implantation technique offers unique advantages in terms of implant stability and interface connection performance. Although the verification of performance changes under long-term movement is insufficient and the in vivo displacement of the electrodes and change in electrode material properties after the development of the animals need to be studied, the idea of forming a stable myoelectric interface through the natural growth of muscle tissue still has a considerable reference importance for expanding the electrode implantation technology of other cyborg animals.

### Sensors

The necessity of situational awareness and information transmission in the remote operation of robots is highly important for task completion and human–robot collaboration; therefore, it is essential to install sensors in electronic backpacks or use global visual tracking solutions to collect information, including the posture and orientation of the robot. Subsequently, algorithms should be used to form a closed-loop control system.

#### Onboard miniature camera modules

Rasakatla et al. [82] used WiFi to transmit images from cameras in cyborg cockroach electronic backpacks to computers, and the operator controlled the robot according to the images; moreover, the use of thermal and visible light cameras can enable human detection in rescue tasks [83]. Further integrating a histogram of oriented gradients and a support vector machine image classification model can achieve real-time human detection with low power consumption, with an average accuracy of 87% and a 95-ms calculation time [86]. However, the above solutions rely on external motion capture systems for positioning and do not solve the depth perception problem from the perspective of insects. Therefore, Li et al. [84] developed a navigation and obstacle avoidance algorithm based on a single camera for cyborg insects. Obstacle avoidance instructions were generated via the weighted sum method, and the navigation success rate reached 73.3% through an unsupervised depth estimation model trained on insect perspective datasets.

As for the onboard miniature camera, continuous iterative training was performed on the cloud without local training, complex preprocessing, and model expansion. These unceasing updates can gradually improve recognition accuracy with lower consumption.

#### Outdoor positioning modules

The outdoor test is necessary to check the system effect and the feasibility of cyborg animal applications; however, the existing outdoor test relies on cameras or observation with human eyes, and long-distance tests with GPS are less frequently used. Conducting long-distance flying tests of cyborg pigeons is important to verify the effect of remote control, and low-power GPS modules such as ATK-NEO-6M [160], SR-92 [49], and ATGM336H-5N [138] are more commonly used. They have submeter positioning accuracy and high update frequency and use the same data format and serial communication, which make it easy to connect with mainstream microcontrollers or embedded systems.

To address the gap between large-scale GPS ceramic antennas and lightweight wireless use, surface mount components such as ceramic chip antennas with a weight of only 1.3 g [61] can be an effective solution. Meanwhile, some new positioning modules support both GPS and Beidou positioning, as well as global navigation satellite system positioning, and they have advantages in terms of size, installation density, and ease of welding assembly.

#### Attitude sensor modules

A highly composite IMU is widely adopted to track the attitude of cyborg animals, and state data from sensors can further participate in real-time and closed-loop control. Sato et al. [62,68] combined IMUs with high-speed cameras, achieving the flight tracking and tilt control of beetles. Xu et al. [34] developed a wearable closed-loop control system for cyborg rats that integrates IMUs and infrared thermal sensors, achieving a search success rate of up to 65% in complex terrains. Yang et al. [30] developed a control system including an exoskeleton and an IMU module, and the IMU is used for heading angle detection and assisting the exoskeleton in positioning. The cyborg rat can be guided to move along the designated path with a positioning error of less than 4%. The prediction feedback control navigation algorithm based on an IMU can immediately apply an acceleration stimulus to keep the cyborg cockroach moving continuously when the speed drops below a threshold, improving the passage of obstacles while decreasing the mission finish time [86]. The online classifier based on IMU signals and feedback incentives to automatically apply stimuli to stagnant cyborg cockroaches significantly increases the search rate and movement distance. This machine learning-based automatic stimulation feedback signal reduces the stimulation amplitude while ensuring control effectiveness and avoiding muscle damage and fatigue [85].

The primary challenge that needs to be addressed is the jitter and noise errors caused by the interaction of animals and backpacks; however, aging and drift in long-term experiments, regular inspection, and calibration will markedly reduce the error. In addition, a dynamic adaptive calibration algorithm and hardware-level optimization, including readout circuit optimization and package systems, may be useful.

### Energy supplement for electronic backpacks

The limitations of animal body size and load capacity, as well as the need for long-term effective remote control, make energy the main obstacle affecting the application scope and duration of cyborg animals. Therefore, the design of low-power electronic backpacks and increasing energy sources are the main research directions for the future.

Recently, polymer lithium-ion solid-state batteries have become the mainstream power supply components for electronic backpacks. However, the low endurance of small batteries hinders the realization of remote control for cyborg animals. To this end, Bozkurt et al. [54,116] designed a superregenerative receiver architecture that can eliminate additional components in conventional superheterodyne topologies and consume less power because of the self-oscillatory and self-quenching advantages of the “superregeneration” principle with only 750 μW static power consumption and only 1 mW dynamic power consumption. The superregenerative architecture is vulnerable to RF leakage out of the antenna and does not support arbitrary squaring and integration intervals. Therefore, Daly et al. [161] designed a noncoherent receiver composed of noncoherent pulsed ultrawideband receivers and pulse-width modulation stimulators using a multistage inverter-based RF front end with a resonant load and differential signal chain, achieving stable and low-power operation. The receiver power is only 8.38 mW when the data rate is 16 Mb/s.

**Fig. 10.**
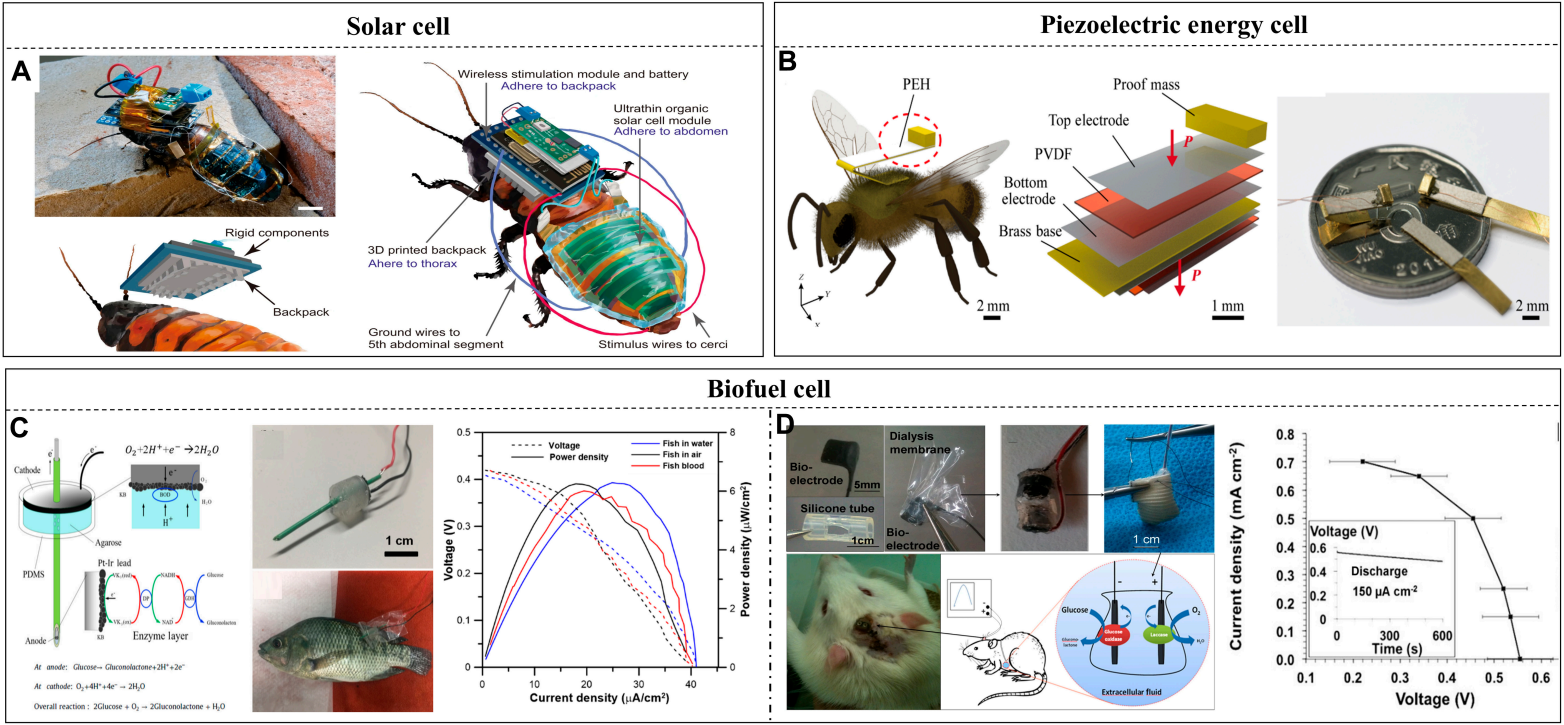
Energy harvesting methods for cyborg animals. (A) The cyborg cockroach uses a thin solar film to harvest solar energy. Reproduced with permission [163]. Copyright 2022 Springer Nature. (B) The bee uses a piezoelectric energy harvester to harvest vibrational energy. Reproduced with permission [166]. Copyright 2025 AAAS. (C) Enzymatic biofuel cells are implanted into the caudal area of living fish to access biofuels. Reproduced with permission [170]. Copyright 2019 MDPI. (D) Bioenergy harvested from rat body fluids. Reproduced with permission [172]. Copyright 2013 Springer Nature.

Collecting external energy to power an electronic backpack is an effective measure for enhancing endurance. The 20-mAh battery can be fully charged by solar panels in 2 h under a focused white LED [162]. Kakei et al. [163] developed a flexible solar panel composed of ultrathin solar panels and adhesive–nonadhesive interleaving structures (Fig. 10A), which can achieve high power output and insect self-righting, and the endurance of the electronic backpack of cyborg pigeons with solar panels can be increased up to 148% [39]. In addition, several energy-harvesting methods have been developed with a certain power output capacity; however, few can be combined with electronic backpacks. Thermal energy harvesters can be used to harvest the temperature difference energy between the body of the animal and the surrounding air [164], while piezoelectric energy harvesters can collect energy from the vibration of the thorax during insect and bird flight (Fig. 10B) [165,166], from the tail swing of a fish while swimming [167], and from the amplitude changes of the body during rat movement [168].

In addition, biofuel cells, which can directly convert chemical energy into electrical energy using biological catalysts or biomass fuels, have been studied extensively [169]. The maximum power density of an enzymatic biofuel cell in tilapia is 8.6 μW/cm^2^ [170] (Fig. 10C). For generating electricity via trehalose in the hemolymph of insects, the maximum power density can reach up to 55 μW/cm^2^ [171]. A glucose fuel cell implanted in a cyborg rat can achieve a peak power density up to 193.5 μW/cm^2^ under physiological conditions (Fig. 10D) [172]. The cyborg cockroach developed by Shoji et al. [173], which uses the animal’s energy, can also collect environmental information through onboard sensors and may be the most practically applicable cyborg animal. However, the influence and exploration of the locomotion ability, stability, and animal health remain lacking, and further research is required.

In the future, in addition to combining different energy harvesting methods with electronic backpacks, advanced technologies such as triboelectric nanogenerators [174] and wearable thermoelectric generators [175] can be utilized to overcome the energy self-supply and energy circulation bottlenecks of cyborg animals.

## Navigation Control for Cyborg Animals

With the achievement of basic controlled locomotion, researchers explored benign changes realized through the intervention of smart algorithms and achieved single and swarm navigation.

### Algorithms to improve locomotion patterns

**Fig. 11.**
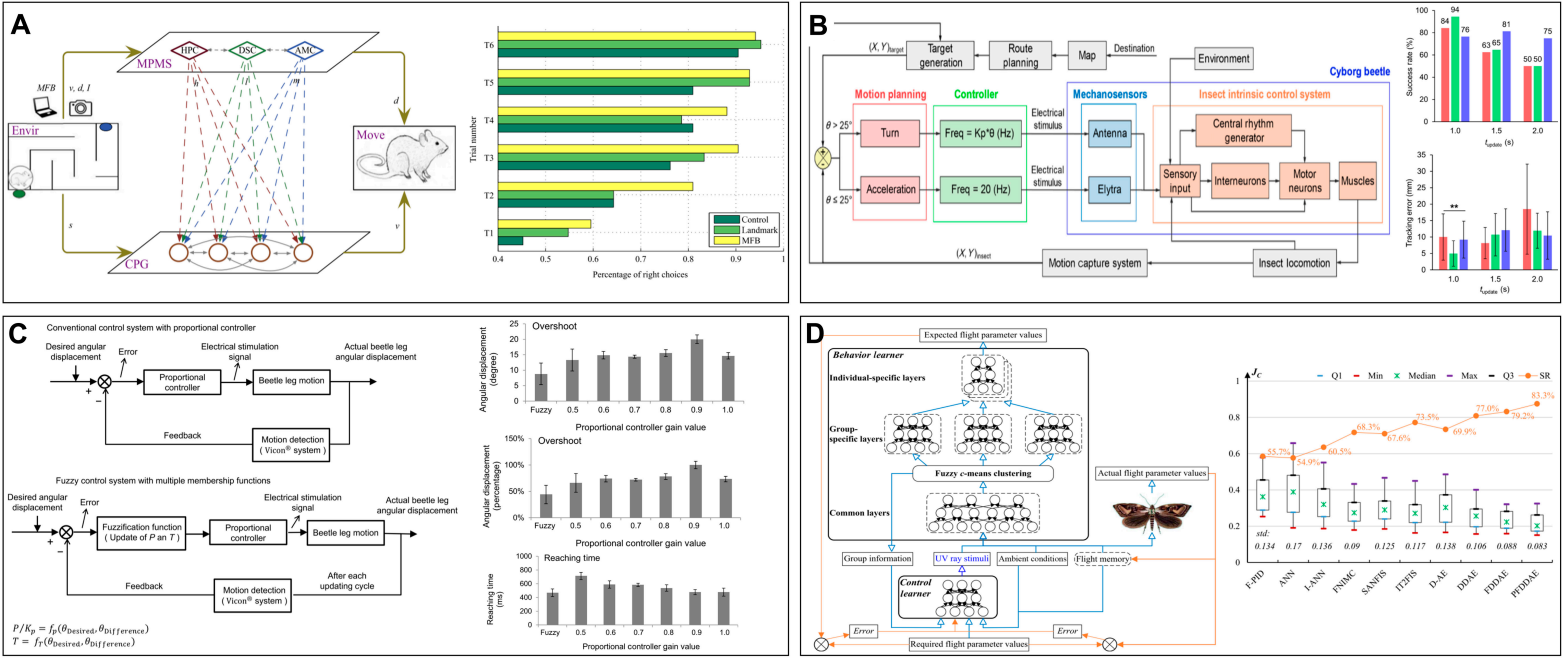
Locomotion patterns adjusting algorithms of cyborg animals. (A) Hybrid control algorithm framework based on the MPMS system and CPG motion generation model can forecast the locomotion of the cyborg rat with a relatively high accuracy rate. Reproduced with permission [176]. Copyright 2015 Elsevier. (B) Hierarchical feedback control algorithm for the cyborg beetle can lead a low-error tracking navigation. Reproduced with permission [77]. Copyright 2023 Elsevier. (C) The fuzzy control system with multiple membership functions is beneficial for reducing overshoot and the arrival time of path locomotion. Reproduced with permission [76]. Copyright 2016 Elsevier. (D) The performance (in terms of regression error) of the cyborg moth flight control algorithm based on fuzzy deep learning is the best among several algorithms. Reproduced with permission [178]. Copyright 2022 MDPI.

Closed-loop control algorithms are critical for increasing the control success rate and stability of cyborg animals. These algorithms adjust the system output through a feedback mechanism to reach the target. The hybrid biomachine multimodal memory system (MPMS) and CPG network model simulating quadruped gaits based on a Wilson–Cowan oscillator and combining a biological memory system with an electromechanical control system as an MPMS-CPG hierarchical architecture computing model can predict cyborg rat movement patterns and improve the speed of behavioral learning; however, the lack of testing in different scenes makes the real effect remain unknown (Fig. 11A) [176]. The enhancement of the autonomy of cyborg rats through machine learning and neural regulation with human operation motion parameters (such as position, direction, and offset angle) and corresponding instructions as model inputs and using a general regression neural network to learn control logic can automatically generate instructions when controlling the movement of the rat. The success rate and accuracy of machine intelligent control were similar to those of manual control, effectively reducing the burden of manual operations [31]. An algorithm with stimulation parameter adjustment and reactivation functions for cyborg cockroaches can significantly extend control duration and ensure control accuracy in response to different scenarios [177].

The insect leg movement control system based on fuzzy control for the crawling navigation of cyborg beetles can adapt to the nonlinearity and time-varying characteristics of biological muscles through multimembership functions based on leg movement angles and achieve movement path tracking by precisely adjusting the frequency of muscle electrical stimulation, significantly reducing overshoot and adjustment time compared to that with traditional PID control (Fig. 11C) [76]. The success rate of path tracking navigation can reach 94%, and path tracking accuracy of only 1/2 of the body length by integrating proportional and thrust controllers (Fig. 11B) [77]. Yang et al. [178] proposed a noninvasive flight control method for cyborg moths based on a Pythagorean fuzzy deep denoising autoencoder, constructing a hierarchical control model through 34-dimensional environmental parameters and historical flight data. They used the improved possibility fuzzy c-means algorithm to learn species-general behavior, group-specific behavior, and individual-specific behavior (Fig. 11D). Eventually, this system achieved an 83% control success rate with an output deviation of less than 15%. This method can help reduce the uncertainty caused by individual differences. Further verification of the generalization under strong wind/outdoor disturbances can enable the construction of a complete test system.

The most important factor in improving the locomotion patterns of cyborg animals is the adaptation to individual differences and stimulation habituation. Control problems arising from individual differences can be mitigated either by leveraging the autonomous movement behaviors of animals as much as possible and applying stimulation only at key position nodes along the path or by alternating stimulation sites and varying stimulation parameters, thereby prioritizing the use of goal-oriented control methods instead of strictly controlling each action.

### Navigation algorithms

The navigation effect is one of the most important ways to test the locomotion ability and adaptation of cyborg animals. Typically, path planning, identification and positioning, feedback control, and AI decision models are all included, and planning methods and decision preferences play an important role in influencing the navigation process.

The intelligent navigation system for cyborg rats that integrates real-time trajectory information from a bird’s-eye camera and map can derive stimulation control commands from motion state data and preset navigation plans and finally transmit the commands to the stimulator. This helps achieve closed-loop automation from video capture to detection, decision-making, and stimulus control; however, no quantitative success rate and error analysis is conducted [29]. For real-time computer-assisted enhancement of cyborg rat navigation, stimulus control is performed using a computer with an overhead camera to achieve an optimal navigation path. The control effect is better than that of free rats and fully controlled cyborg rats; the steps, path length, and total time decreased significantly [32]. Ariyanto et al. [136] proposed 2 sets of algorithms for different density obstacles: reach-avoid navigation and adaptive reach-avoid navigation, which coupled arrival avoidance and free walking; the highest navigation accomplishment rate is 69.23%, and the least final deviation is 3.89 cm (0.6 of the body length) during the tests in granular and rocky soft terrain with external Vicon devices for positioning.

**Fig. 12.**
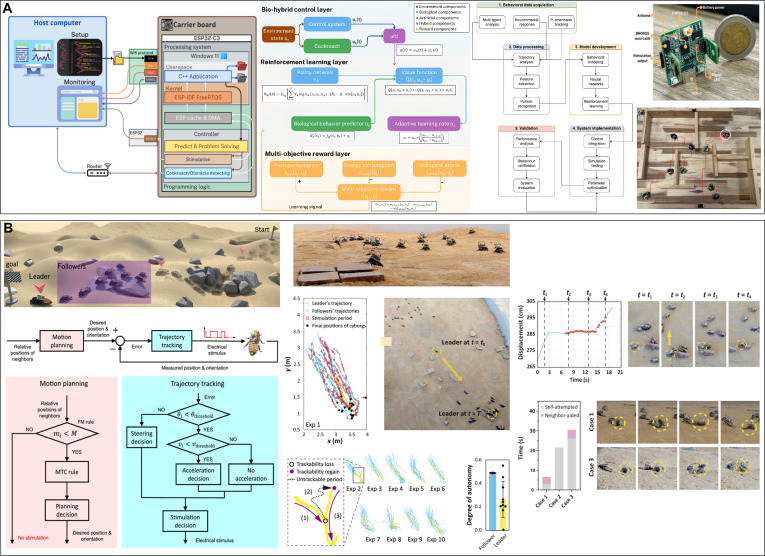
Cyborg animal swarm control algorithm. (A) The behavior modeling and enhancement of swarm cyborg cockroaches based on reinforcement learning (RL) can lead to the group’s own higher cohesion, consistency, and efficiency. Reproduced with permission [179]. Copyright 2025 IEEE. (B) The swarm cyborg cockroaches guided by the tour group inspired (TGI) algorithm can improve travel efficiency, reduce stimulation frequency and entanglements, and can even improve the ability to recover from overturn through the support points provided by the neighbors. Reproduced with permission [180]. Copyright 2025 Springer Nature.

Residual reinforcement learning with Q-learning and the pause mechanism can be used for single and swarm navigation; the completion speed is 57% faster than that of natural cockroaches. The trajectory variance decreased by 72% in maze missions, while the pause mechanism reduces the occurrence of stimulating events by 62% (≈4.8 times per minute) (Fig. 12A) [179]. In addition, the framework for cyborg cockroach swarm navigation in unknown, obstructed, and soft terrains proposed by Bai et al. adopts the tour group inspired (TGI) algorithm that contains the free motion rules and move-toward-crowd rules. The group is divided into the leader and the follower, and only the leader is aware of the target location. Each individual only perceives and distinguishes neighbors within a limited perceptual range and differentiates between the leader and follower roles. Motion planning and trajectory tracking can induce movement through less powerful stimulation strategies. The experiment used 1 leader and 19 followers, and the results showed that the degree of autonomy was higher, the stimulation number was lower, and the interactions among neighbors helped each other get out of trouble and recover from an overturned posture (Fig. 12B) [180].

In the next step of the control algorithm for cyborg animals, replacing external positioning with onboard self-positioning and relative positioning, and the use of geometric path tracking and other tracking methods can promote the more precise formation of remote location and control. Meanwhile, to better deal with sudden and complex scenarios within long-distance missions, the low-cost and quick-response intelligent decision-making algorithms are important, and model optimization, pruning, and a lightweight software framework can be considered. Further, the group behaviors of animals have a high utilization value for wide-range environmental detection and collaborative tasks; therefore, adding multiagent reinforcement learning and graph attention networks and other learning algorithms can further improve the level of swarm coordination and robustness. Finally, swarm cyborg animals with stable and strong swarms and excellent mission execution and completion can be constructed.

## Application and Development

### Practical techniques for application

Exploring more practical techniques is crucial for promoting their application while making breakthroughs in the fundamental technologies of cyborg animal locomotion control.

#### Animal ability expansion

**Fig. 13.**
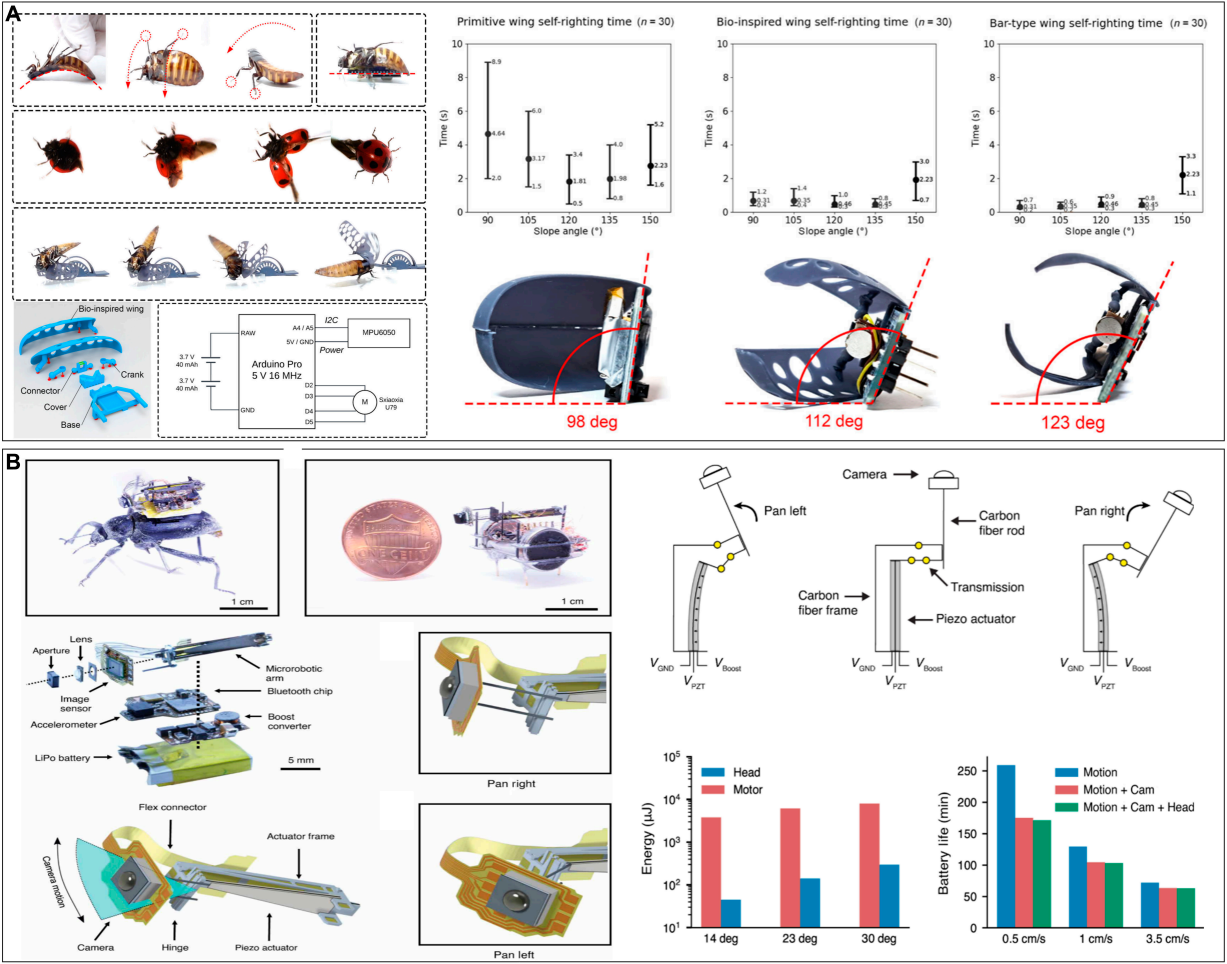
Examples of cyborg animal ability expansion. (A) The coccinellidae-inspired 3D-printed artificial elytra mounted on the cockroach can offset the impact force and grant it self-righting ability upon landing. Reproduced with permission [182]. Copyright 2024 Springer Nature. (B) Mini-camera based on a low-power piezoelectric drive combined with the beetle’s vision can provide higher image resolution, energy efficiency, and endurance than a wide-angle lens that covers the same field of view. Reproduced with permission [183]. Copyright 2025 AAAS.

Animals are not perfect, and the absence of locomotion functions and inherent weaknesses may affect overall ability; therefore, the ability expansion is meaningful to cyborg animal development. Assistive devices developed to address the physical defects and insufficient movement functions of animals can enhance or expand the movement and perception capabilities of cyborg animals. For example, replacing the natural leg spine with an artificial leg spine can enable a cyborg beetle to walk backward [181]; the exoskeleton EL designed on the basis of the EL of ladybugs can increase the success rate of self-righting the cyborg cockroach (Fig. 13A) [182]; and the mini-camera driven by a microactuator mounted on the head of the cyborg beetle can expand its field of view (Fig. 13B) [183].

#### Control of activity range

The virtual fencing technique does not use physical barriers and instead controls the movement of animals via external stimuli to drive the animals back when they are outside the electronic boundary defined by the GPS and geographic information system, achieving activity range control [184]. For example, Latif et al. [162] designed a virtual fencing system to keep cyborg cockroaches within a controlled range and drive them toward a light source to charge an electronic backpack. This technique offers a dynamically adjustable working area that can enhance management efficiency, especially for controlling swarm robots.

#### Batch manufacturing

The development of each cyborg animal involves customized operations because of individual differences among animals and the various task and functional requirements, which requires highly skilled manufacturers and is time-consuming. To address this issue, Lin et al. [185] developed an automated cyborg cockroach electrode implantation system that uses a deep learning-based visual system for identifying electrode implantation reference points and automatically implanting electrodes. Compared with manual implantation, the operation time was reduced from more than 1 h to ≈8 min, and the consistency of the implantation effect was ensured simultaneously, providing an important reference for the future large-scale production of cyborg animals.

### Applications

Although cyborg animals have not been widely applied, explorations, including environmental detection based on cluster control, post-disaster search and rescue, and human–computer interaction writing, have already begun.

#### Cluster control and environmental detection

Environmental detection in vast regions requires the cyborg animal team to be under cluster control, providing a foundation for efficient exploration. The application of cluster-controlled cyborg animals not only includes environmental detection and searching but also is meaningful for the hybrid interaction of humans, cyborg animals, and traditional robots.

A single-agent inertial navigation system can achieve centimeter-level positioning in a non-GPS environment using a data-driven inertial navigation system [186]. The use of proximity information among robot agents can increase the accuracy of trajectory reconstruction [187], and the swarm navigation algorithm can combine the free movement of insects with the group attraction rule to induce efficient cooperative navigation in complex terrains [180]. In addition, a robot coordinate and environment mapping framework based on topological data analysis can extract environmental topological features from random encounter events to construct topological maps [188] and achieve dynamic stitching of local maps and mapping of global maps [189]. A distributed sensor network constructed based on the sound source localization capability of the onboard microphone array of cyborg cockroaches can provide multinode signal transmission capabilities [190–192], significantly affecting the detection and rescue of survivors in earthquake ruins and other narrow environments. In March 2025, 10 cyborg cockroaches with thermal imaging cameras and navigation sensors conducted the world’s first actual disaster relief operations in Myanmar and took a solid step forward in practical application [193]. After the gold nanorods of the plasmonic nanotattoo for cyborg locusts are functionalized, changes in subsequent surface-enhanced Raman scattering can be detected and used for explosive detection [91].

#### Control of human–machine interactions

**Fig. 14.**
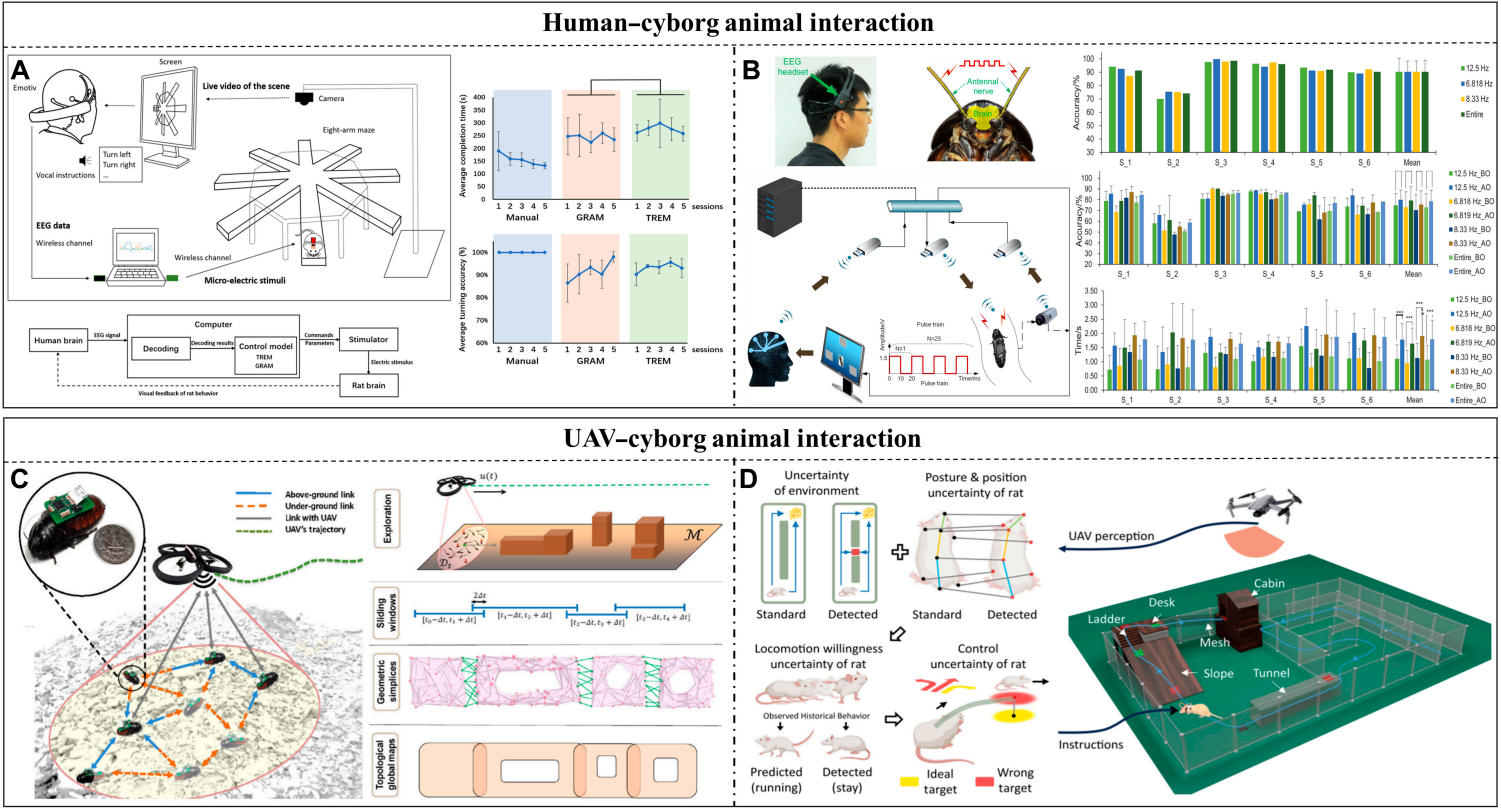
Examples of interaction and cooperation between organism and robot. (A) The interaction that integrated electroencephalogram-based motor imagery and brain stimulation can realize the locomotion of the cyborg rat through human mind. Reproduced with permission [196]. Copyright 2019 Springer Nature. (B) Human motion intention can be recognized through SSVEP-based BCI and then convert to the electrical stimulation command to guide the cyborg cockroach move along a S-shape track. Reproduced with permission [195]. Copyright 2016 PLOS. (C) Combining the movement of the cyborg cockroach with the UAV leader, the global map can be merged through the topological characteristics of the local map constructed from the encounter information of the cyborg cockroach. Reproduced with permission [189]. Copyright 2017 Elsevier. (D) The collaborative Rat-UAV navigation (RUN) paradigm can dynamically plan paths with partial observations from UAV and stably control the cyborg rat navigation in the maze. Reproduced with permission [198]. Copyright 2024 IEEE.

Human–machine interaction is a novel approach to establish a functional interface between a human operator and a cyborg animal, and this can lead to the development of BCI techniques and man-in-the-loop control methods. By using steady-state visually evoked potentials and BCI technology, neural signals from the human brain can be translated into electrical stimulation commands for animal BCI controllers. For example, stimulating the nigrostriatal of rats can help control their navigation [194], while the electrical stimulation of the AT of cyborg cockroaches can help control their movement (Fig. 14B) [195]. Combining the motor imagery BCI with brain stimulation can enable precise control of the continuous movement of cyborg rats in 3D mazes by humans (Fig. 14A) [196,197].

### Cooperation between cyborg animal and unmanned robot

Automation and intelligence are currently popular concepts in technological development, and cooperation between cyborg animals and unmanned robots can provide a new possibility for developing a hybrid robot system. The combination of adaptability, animal intelligence, and low-cost unmanned robots can aid in constructing a highly practical novel hybrid robot paradigm. For example, the combination of cyborg cockroaches and unmanned aerial vehicles can explore, gather, and map the complex area; then, the geometrical and topological global map can be formed from the local map messages (Fig. 14C) [189], in addition to using the bird’s-eye view perspective of the UAV and modeling, in addition to decisions based on dynamic Markov decision processes, which enables cyborg rats to navigate in complex environments, thereby providing a feasible solution for animal–machine collaborative navigation (Fig. 14D) [198].

### Future applications

The maturity and completeness of cyborg animals will continue to improve with the continuous development of related technologies, including control, communication, and energy. The deployment of different cyborg animal nodes in multidimensional environments, including land, aquarium, and sky environments; interconnection with multiple cyborg animals through wireless neural interfaces; and distributed AI networks in a multidimensional and multilevel intelligent perception and action system can be performed, and the body characteristics of animals at different scales can be utilized to achieve multiscale coverage. For example, centimeter-scale cyborg insects can penetrate microcracks, meter-scale rodent robots can construct cave networks, and cyborg fish can patrol rivers, enabling seamless coverage from the microscale to the macroscale. Simultaneously, the most appropriate robot nodes can be dispatched through network collaboration to achieve task adaptability and optimization by leveraging the special sensing abilities of different animals, such as the sensitivity of insects to odors and the sensitivity of rats to subtle vibrations. In addition, by exploiting the low environmental interference and natural adaptability of cyborg animals as well as their ability to operate without charging and long-term cruising, the sensor information gathered by robots can be integrated into a time-series statistical parameter system through a cloud data platform to perform continuous pollution source detection, climate change monitoring, biodiversity research, and other environmental exploration and ecological protection tasks with minimal impact on ecological balance and wildlife habitats. They can explore dense jungles, unknown underground water systems, and other narrow spaces that are difficult for humans and traditional robots to access and map outdoor ecological information. In the event of sudden scenarios such as the invasive species or element being over limited in a certain area, specific cyborg animals can be dispatched to achieve “flexible” regional purification or biological removal. The role of cyborg animals in field rescue and disaster relief is similar to that of “life probes”. They can search for survivors trapped in narrow or dangerous spaces with themselves and devices, including heartbeat sensors, carbon dioxide sensors, and sound sensors in electronic backpacks. In battlefields or environments with nuclear radiation, chemical pollution, or other harsh conditions, animals selected or genetically modified for tolerance can be transformed into cyborgs to perform tasks such as explosive detection, special reconnaissance, and intervention, instead of humans. Cyborg animals can achieve rapid deployment, retrieval, and dynamic strategy adjustment because of the distinctive advantages of animals, including their small size, high concealment, easy deployment, no need for complex logistical maintenance, and ability to seek benefits and avoid harm. A closed-loop system for crop pollination and pest and disease monitoring, prevention, and control can be constructed by combining small cyborg animals with other microrobots. These animals can conduct fine and high-frequency inspections and provide pollination or local spraying of trace amounts of drugs for key crops through onboard miniature cameras and drug- or pollen-release devices as required because of the natural and excellent flight capabilities of insects and birds, thereby achieving precise prevention and control with zero chemical residues while saving labor costs and improving efficiency.

Compared with traditional silicon-based robots, animals possess tissue flexibility, self-repairing capabilities, and adaptability to complex environments, and living cyborg animals can complete actions and missions difficult for traditional robots to achieve (such as traversing jungles, deep-sea swimming, and high-altitude flight) without complex mechanical structures. Further, animal perception systems are far superior to general sensors because they have various types of receptors distributed throughout their bodies with outstanding perception accuracy, density, and response capabilities; different receptors can quickly integrate and analyze different external stimuli to make fine adjustments. Therefore, living cyborg animals do not require additional sensors and have excellent perception and response capabilities. The retention of the brain enables cyborg animals to still possess excellent behavioral decision-making abilities and the ability to seek benefits and avoid harm, thereby preserving their natural advantages in completing complex continuous tasks. Meanwhile, the energy conversion efficiency and endurance of cyborg animals far exceed those of battery-driven robots and have become more suitable for long-term activities when the energy can completely rely on themselves.

### Development and challenge

Cyborg animals are complex systems involved in disciplines such as sports biomechanics and robotics. Finding a balance between biological intelligence and machine intelligence is necessary. A new type of biorobot constructed by considering the strengths of both has special advantages for performing complex tasks and making autonomous decisions. However, most relevant studies are currently in the laboratory stage, and the long-term stability and biocompatibility of many technologies are insufficient for large-scale applications.

For control, the primary constraint on the enhancement of robotic performance pertains to the generalizability of stimulus parameters. The efficacy and outcomes of control strategies for animals of the same species and under identical conditions differ because of the inherent variability among individual animals, the same electronic backpacks, and the control strategy for cyborg animals. The successful control rates and performances of individuals are obviously different, and they even show several-fold gaps not only between different individuals but also in different trials of the same individual [85]. Consequently, developing control models that can universally control cyborgs of the same species without the problem of consistency among animals is necessary. This model can ensure 100% task completion while not requiring the execution process of each robot to be completely consistent. This simplifies research and development processes and reduces the difficulty of control.

In addition, there is a decision conflict or execution priority problem between the stimulus commands and animals themselves, i.e., a compatibility problem between animal and machine intelligence. Regardless of the control strategy, animals invariably exhibit behaviors that deviate from their intended strategies. A state of hesitation occurs when the natural movements of the cyborg animals are conflicted with stimulus control sequences, particularly in contexts where food attraction or natural enemies exist. For example, the cyborg pigeon turns to the ipsilateral side when its SGP brain region is stimulated normally; however, in a relatively small number of cases, it turns to the contralateral side, and the rate of being absolutely wrong is ≈9% according to the experimental data [199]. Then, the achievement and efficacy of the control strategy can be doubted; therefore, it is crucial to retain and utilize the inherent tendency of the animals to avoid harm. A topic designated the “biology-machinery game” has become a pivotal research subject to ensure control effectiveness without interfering with the natural behaviors of animals. As a solution, a fault prediction algorithm can be used to prevent conflicts between internal behaviors and navigation commands by monitoring insect movements [86].

There are defects in quantifying animal behavioral scales and control parameters. Existing cyborg animals have demonstrated the capacity to execute behavioral hierarchical control; however, they still lack the ability to utilize encoders to align pulse fluctuations with the steering angle and other parameter functionalities as traditional robots do. The most common description is the reaction scale or reaction time related to commands. This phenomenon can be attributed to the issue of differentiating the control effect, which is influenced by interindividual variability and interference of neural signals. Therefore, the importance of future studies to clarify the mechanism of multiparameter signaling collaboration and coordination to achieve precise control and quantify key metrics, including the speed and distance of the robots in relation to the output frequency of the muscle group, magnitude of torque, and other parameters, is self-evident.

The simplicity of the stimulus-inspired actions of cyborg animals is an issue. Currently, robots performing actions in accordance with specific stimulus parameters can only be executed through a single instruction that defines a specific spatial plane of action, and they have a low degree of action combination capability. A relatively successfully controlled cyborg animal 3D motion was achieved through BCI-based cyborg rats with rough control that requires frequent human intervention [7]. Further, it is harder for muscle stimulation control and other techniques not only to achieve complex movements but also to ensure simple obstacle avoidance and crossing. Excessive movements are also usual, and frequent adjustments are required. Therefore, developing control algorithms that combine multimuscle group-coordinated stimulation and feedback loops can help realize more complex actions, such as hovering and saccades. Simultaneously, a combination of preprogrammed stimulation sequences and motion state processors (such as GPS timing) [200] can enable the controller to automatically invoke corresponding subroutines at a predetermined position or posture and achieve real-time autonomous regulation. Simultaneously, there may be solutions from neuroscience, especially for BCI-based cyborg animals that are almost exclusively focused on control through the emotion region of the brain, making it difficult to adjust the action phase and accuracy and exhibit a relatively higher delay. Therefore, closed-loop feedback control based on sensory-motor integration and decomposed control methods of the brain and behavior performance of the muscles or the spinal cord [201] can provide inspiration for future control thought.

For the design of electronic backpacks, the load on animals has an important effect on their energy consumption, movement efficiency, speed, endurance, and other performances. For example, a load exceeding 5% of birds’ weight severely affects their flight [140], and it is valuable to integrate, miniaturize, and reduce the weight of the electronic backpacks. The dual reduction in size and weight can be achieved while maintaining performance by selecting a highly integrated SoC/MCU and designing dedicated ASIC chips to integrate sensor signal conditioning (amplification and filtering) and ADC functions. Further, using microsized PCBs and 3D packaging technology to reduce size, adopting a wireless power supply or energy harvesting technology to power sensors and reduce wiring, simplifying communication protocols, and reducing redundancy and data validation can help optimize performance. Using flexible electronic devices [202], skin-like sensors [203], and other flatter and skin-friendly components can improve the long-term reliability and biocompatibility of electronic devices, thereby reducing the discomfort and impact on the movement flexibility of cyborg animals. In addition, the outermost packaging, including the LCP [25] and PCB based on the stiffness-adjustable temperature-responsive ink [204], can further improve the flexibility and adaptation of implantable embedded systems, respectively.

Overcoming the difficulties of insufficient long-term control stability and stimulation success rates and avoiding interference and damage caused by the diffusion of stimulation current in the tissue is essential when designing and implanting stimulators. For example, the successful eclosion rate of moths after EMIT is 90%, and some adults fail to spread their wings properly [157]. Precise stimulation can be achieved through low-impedance and highly biocompatible flexible electrodes or targeted stimulation, and the multimodal control of behavioral combinations can be achieved through multichannel stimulator arrays. The development of miniaturized high-density electrode arrays, directional current-focusing, current compensation, and frequency-encoding modulation technologies are beneficial for achieving selective muscle activation and improving the spatial resolution of stimulation. Tissue damage and fibrosis caused by the modulus mismatch influence the efficiency and stability of microelectrodes. Novel materials and techniques can further improve performance and biocompatibility from the electrode manufacturing perspective; e.g., bioelectrodes based on conductive hydrogels [205] are conducive to the minimally invasive injection shaping and high conductivity. The electrodes made of liquid metal can achieve ultrahigh compliance and high-density graphitization, and they are compatible with large deformation environments [206]. The packaging of electrodes is also important, especially the reliability maintained under the coupling conditions of a high-salt environment with reactive oxygen species and low-intensity exercise, such as the multilayer immersion coating for preventing corrosion and contamination and maintaining impedance and stability, material passivation for reducing ion permeation, and the adaptive base and buffer layer for adapting to the minor fluctuating scenarios. In addition, the difficulty and time required for implantation also need to be overcome, considering insects as an example. The basic surgery time of one cockroach is at least 15 min [124]. More complex animals and more precise implantation cost more time. Further, it is necessary to address the problem of habituation that animals tend to develop when suffering from long-term stimulation, no reaction, or unforecasted illegal reactions. The incomplete movements happen because repeated stimulation leads to incomplete recovery from the refractory period and muscle fatigue, or the reaction decline caused by the ineffective stimulation parameters. To address these issues, the constant change of signal patterns [177] and incorporation of break windows can be used. No reaction is likely to occur after long-term stimulation. If there is no hardware or software fault, the target tissues are already damaged or obviously fatigued, and as an absolute or relative inactive period, forced rest, reduced stimulation intensity, and changed stimulation sites should be applied to reduce animal stress and injury. Further, real-time or long-term monitoring and inspection are necessary to consider the health conditions of animals and ensure the effectiveness of animal–machine interfaces.

In terms of communication and positioning techniques, existing onboard modules have a communication distance of ≈100 m. Some backpacks can only be used within 10 m, and they are all difficult to use practically. Therefore, developing communication and positioning technologies with long communication distances, strong anti-interference performance, and low energy consumption for cyborg animals is essential in relatively closed and narrow working scenarios or in those with strong interference, such as communication network technology based on multinode transmission [191], real-time kinematic technology, and geomagnetic positioning technology. Further, integrating and exploiting biological and mechanical electronic sensing to construct a complete, high-precision, and rapid-response perception system with only a few additional sensors can help leverage the advantages of animals and enhance the ability of cyborg animals to work independently and autonomously in complex environments.

Using cluster control and cooperation to explore the area more efficiently and achieve more functions is an important future research direction, and it can be divided into exploration and collaboration. The set of cluster control exploration strategies determines the effect and efficiency, and the TGI algorithm proposed by Bai et al. [180] reduces the possibility of robot entanglement and increases the free movement time, whereas the main mission is swarm navigation from the start to the end point. In the future, regional or task-based control methods and adaptive and self-learning techniques can be used for improving cluster control. A multirobot autonomous exploration method based on the transformer model can be adopted to improve exploration efficiency [207], and a decentralized method can be adopted to optimize the process of information sharing [208]. Task allocation is the essence of collaboration, detailed task planning is conducive to completion, and a different algorithm can be adopted for a different target, including reducing the cost of the task, maximizing the gains, and so on [209].

Ultimately, overcoming the challenges associated with the large-scale production of cyborg animals and designing interactive hybrid robot systems of cyborg animals, bionic robots, and humans can help provide innovative patterns to explore new organism–machine interaction modes [210,211] and deep multilevel environmental detection, including the deep sea, battlefields, and high-risk and high-pollution areas.

### Ethical considerations for laboratory animals

Ethical considerations for laboratory animals are important and investigated further. The existing articles declare that animal studies have been approved by the ethics committee of their college or research institution, and they indicate that all operations are compliant with research guidelines, such as the Regulations of China’s Guide for the Management of Laboratory Animals [48], to minimize animal suffering and provide the messages of the animal they used, including breeds, resources, and information on how to keep animals healthy during the experimental period.

In international and national standards, animal welfare must be placed at the forefront of experimental design, and the principle of the 3Rs (replacement, reduction, and refinement) has been the bottom line of review and supervision [212]. During these studies, high-fidelity simulations or deceased individuals were used for replacement, decreasing the number of experimental animals, optimizing surgical processes and stimulation values, and adopting other measures to treat animals kindly. Further, the humane end points are important, which include “manually” determining an experiment termination point (or stage) without affecting the judgment of the experimental results, including excessive pain, inability to survive normally, being on the verge of death, or inability to move. Human intervention can help minimize the pain without sacrificing scientific validity. Meanwhile, body structure and surgery vary from one species to another; therefore, different ethical considerations must be considered. Vertebrates have been covered by strict ethical regulations and require formal ethical clearance from an institutional animal care and use committee or equivalent body; however, there are fewer regulatory bodies for insects and other animals. Although it is believed that insects do not experience pain or suffering in the same way as more complex organisms, standardized procedures and animal management are still essential.

The strict constraints on animal experiments are not intended to hinder scientific progress but rather to strike a prudent balance between promoting scientific advancement and fulfilling moral responsibilities. We share our ability to perceive the world with animals; therefore, it is necessary to assume the responsibility of caring for the animal. Through laws and regulations, an ethical consensus can be transformed into executable standards to ensure that science progresses on a track that aligns with human values; these constraints are an indispensable part of modern responsible research behavior. Consequently, a well-established welfare maintenance system and a complete regulatory framework can help enhance social approval and trust in cyborg animal research and applications.

## Conclusion

As a paradigm for exploring novel robotic systems that integrate carbon- and silicon-based components, cyborg animals exhibit distinctive advantages in terms of autonomous decision-making, complex environment adaptation, and energy efficiency. Their development not only advances robotic systems but also helps pioneer biomedical technologies and devices. A series of cyborg animal control strategies and related technologies have been developed and adapted to different environments through exhaustive research on control technologies under different stimulation modes and task orientations for different types of animals with different locomotion modes. A cyborg animal framework was preliminarily constructed, and the flexible regulation and control of locomotion modes were realized. However, several challenges remain, including the limitations of a single control mode, suboptimal biocompatibility, inadequate long-term stability, poor intelligent decision-making, and low control levels.

In the future, a more stable, efficient, and friendly organism–machine interface will be established with the intervention of flexible electronics and self-supply of energy techniques with the improvement of biocompatible interfaces and adaptive stimulation. Subsequently, the use of animal–machine hybrid sensing systems, multimodal control, reinforcement learning, and other AI techniques can improve the intelligence and reliability of closed-loop swarm cyborg animal systems, further promoting the scope of practical applications. A consensus on standardized welfare agreements and ethical regulatory frameworks for cyborg animals, including animal surgery processes and care during and after the mission, can simultaneously accelerate the development of domain standardizability and enhance social recognition. The ultimate goal of cyborg animals is to break through the performance limits and operational boundaries of traditional robots, achieving a human–machine–animal hybrid intelligent robot system and providing solutions for long-term exploration and operations in extreme environments.

## Data Availability

No new data were created or analyzed during this study. Data sharing is not applicable to this article.
